# Rho factor mediates flagellum and toxin phase variation and impacts virulence in *Clostridioides difficile*

**DOI:** 10.1371/journal.ppat.1008708

**Published:** 2020-08-12

**Authors:** Dominika Trzilova, Brandon R. Anjuwon-Foster, Dariana Torres Rivera, Rita Tamayo

**Affiliations:** Department of Microbiology and Immunology, University of North Carolina at Chapel Hill School of Medicine, Chapel Hill, North Carolina, United States of America; University of Pittsburgh School of Medicine, UNITED STATES

## Abstract

The intestinal pathogen *Clostridioides difficile* exhibits heterogeneity in motility and toxin production. This phenotypic heterogeneity is achieved through phase variation by site-specific recombination via the DNA recombinase RecV, which reversibly inverts the “flagellar switch” upstream of the *flgB* operon. A *recV* mutation prevents flagellar switch inversion and results in phenotypically locked strains. The orientation of the flagellar switch influences expression of the *flgB* operon post-transcription initiation, but the specific molecular mechanism is unknown. Here, we report the isolation and characterization of spontaneous suppressor mutants in the non-motile, non-toxigenic *recV flg* OFF background that regained motility and toxin production. The restored phenotypes corresponded with increased expression of flagellum and toxin genes. The motile suppressor mutants contained single-nucleotide polymorphisms (SNPs) in *rho*, which encodes the bacterial transcription terminator Rho factor. Analyses using transcriptional reporters indicate that Rho contributes to heterogeneity in flagellar gene expression by preferentially terminating transcription of *flg* OFF mRNA within the 5’ leader sequence. Additionally, Rho is important for initial colonization of the intestine in a mouse model of infection, which may in part be due to the sporulation and growth defects observed in the *rho* mutants. Together these data implicate Rho factor as a regulator of gene expression affecting phase variation of important virulence factors of *C*. *difficile*.

## Author summary

Phenotypic heterogeneity maintained by phase variation allows bacterial subpopulations to overcome potentially detrimental stresses in the environment, contributing to bacterial survival. Phase variation of flagella and toxins in *C*. *difficile* suggests that maintaining heterogeneity of their production may be important for survival and virulence. In this study, we identified Rho as a *trans*-acting factor that mediates the differential gene expression that imparts heterogeneity in flagellum and toxin production. These results reveal a new role for Rho-mediated transcription termination in regulation of gene expression.

## Introduction

*Clostridioides difficile* is a gram-positive, spore-forming anaerobe and the leading cause of antibiotic-associated diarrheal disease [1]. *C*. *difficile* infections (CDI) are currently among the most common hospital-acquired infections and exhibit a high rate of recurrence [2]. Disease is largely mediated by two large glucosylating exotoxins, TcdA and TcdB [3–5]. These toxins inactivate members of the Rho family of GTPases, causing a loss of interaction between the GTPase and its downstream effectors including those controlling the integrity of the actin cytoskeleton [6, 7]. In cell culture, toxin activity results in disruption of tight junctions, cell rounding, and eventually cell death [8, 9]. During infection of a host intestine, the toxins cause disruption of the epithelial barrier, leading to development of diarrheal symptoms, immune cell recruitment, and inflammation [10]. These toxins are also necessary for development of diarrheal disease in animal models of infection [4, 11, 12].

*C*. *difficile* encodes multiple surface appendages that contribute to cell adherence and/or colonization of the intestine, including fibronectin-binding protein Fbp68, type IV pili, and flagella [13–17]. Flagella are required for swimming motility and are also important for adherence to intestinal epithelial cells, colonization, and virulence in animal models of infection [18–20]. Similar to *Bacillus subtilis* and numerous gram-negative bacteria, the flagellar genes are expressed in a hierarchical manner to ensure the coordinated assembly of the complex structure [21–23]. *C*. *difficile* contains flagellar genes in at least four operons [24]. The early stage (*flgB*) flagellar operon includes the gene encoding SigD, a sigma factor essential for expression of late stage flagellar genes [23, 25, 26]. SigD also positively regulates the expression of toxin genes by activating transcription of *tcdR*, which encodes a positive regulator of *tcdA* and *tcdB* expression [23, 27]. Accordingly, factors that regulate expression of the *flgB* operon, such as cyclic diguanylate, impact toxin production via SigD [22, 25]. Other regulators, including Agr, SinR, and RstA, also affect both flagellum and toxin gene expression [28–32]. For example, SinR activates both flagellar and toxin gene expression [29, 30], while RstA acts as a negative regulator of both flagellum and toxin genes [31, 32].

We recently demonstrated the phase variation of flagella and toxins in multiple *C*. *difficile* ribotypes [33, 34]. Phase variation is a process that generates phenotypic heterogeneity in a bacterial population to help ensure the survival of the population as a whole in response to environmental selective pressures [35]. Typically, phase variation modulates the production of surface structures that directly interface with the bacterium’s environment. Several mucosal pathogens use phase variation to promote immune evasion and persistence in the host [36]. Phase variation occurs stochastically and is also fully reversible to maintain heterogeneity [35]. Multiple genetic and epigenetic mechanisms can lead to phase variation. One mechanisms is conservative site-specific DNA recombination, which involves recombinase-mediated inversion of a specific DNA sequence [37]. The invertible sequence typically contains regulatory information, such as a promoter element, that affects downstream gene expression [38]. DNA inversion requires the action of one or multiple serine or tyrosine recombinases that recognize inverted repeats flanking the invertible genetic sequence and catalyze DNA strand exchange that results in inversion of the intervening sequence [37, 39].

Recent work suggests that *C*. *difficile* employs recombination-mediated phase variation to generate an extensive degree of phenotypic heterogeneity. The first biologically characterized example of phase variation involved the cell wall protein V (CwpV) [40, 41]. Two additional potential DNA inversions were identified upstream of genes involved in cyclic diguanylate metabolism [42]. Most recently, high-throughput sequencing was used to identify a total of seven invertible sequences flanked by inverted repeats in the *C*. *difficile* NAP1/B1/027 ribotype strain R20291, and an eighth in several 017 ribotype strains [43, 44]. The identified sequences were shown to undergo inversion, and the sequence upstream of the *cmrRST* operon was confirmed to regulate expression of the downstream genes in a manner consistent with phase variation [44, 45]. We subsequently showed that site-specific recombination also mediates phase variation of flagella and toxins by inversion of a genetic sequence called the “flagellar switch” [33]. The 154 bp flagellar switch is flanked by imperfect inverted repeats and is located upstream of the *flgB* operon. In one orientation, the flagellar switch is permissive for expression of the *flgB* operon, resulting in “*flg* ON” bacteria exhibiting flagellum biosynthesis, swimming motility, and toxin production. Conversely, the inverse orientation reduces *flgB* operon transcription, yielding “*flg* OFF” bacteria that are aflagellate, non-motile, and attenuated for toxin production. Inversion of the flagellar switch requires the site-specific tyrosine recombinase RecV. Mutation of *recV* leads to genotypically and phenotypically “phase locked” strains that no longer undergo phase variation [33]. In contrast to these phase locked mutants, wild-type *C*. *difficile* R20291 in broth culture consists of a heterogeneous population of *flg* ON and OFF bacteria. Notably, RecV is also required for inversion of the *cwpV* switch, as well as two of the other identified invertible sequences including one shown to impact multiple phenotypes including virulence [40, 44, 45].

The canonical regulatory mechanism of phase variation by site-specific recombination involves an invertible promoter element that, when correctly oriented, promotes gene transcription *in cis*. For example, production of fimbriae by *Escherichia coli* and related species is regulated by the invertible fimbrial switch *fimS*, which contains a promoter for the adjacent *fimA* gene that encodes the fimbrial subunit [46, 47]. The flagellar switch in *C*. *difficile* lies in the 5’ untranslated region of the mRNA, between the previously identified σ^A^-dependent promoter and the *flgB* coding sequence. Previous work showed that the flagellar switch does not contain a functional promoter [33]. Instead, regulation occurs post-transcription initiation and involves an unidentified *trans*-acting posttranscriptional regulator specific to *C*. *difficile*.

In this study, we sought to identify the mechanism by which the flagellar switch controls expression of flagellum and toxin genes. Specifically, how does the orientation of the flagellar switch mediate the phase variable expression of the *flgB* operon? To answer this question, we used a non-motile *recV* phase-locked OFF strain in a suppressor analysis to identify factor(s) involved in inhibiting *flgB* operon transcription, swimming motility, and virulence characteristics. We recovered suppressor mutants that retained the flagellar switch in the OFF orientation but gained extragenic mutations that restored swimming motility, toxin production, and expression of flagellum and toxin genes. The extragenic mutations conferring the suppressor phenotypes mapped to *rho*, which encodes the transcription termination factor Rho. Using a series of reporter fusions in *C*. *difficile* with or without an intact *rho* allele, we determined that Rho inhibits transcription from the 5’ leader sequence of *flgB* mRNA with the flagellar switch in the OFF orientation but not the ON orientation. These results suggest that Rho can discriminate between *flg* ON and *flg* OFF mRNA to selectively inhibit transcription in flagellar phase OFF variants, and reveal a role for Rho-mediated transcription termination phase variation of *C*. *difficile* flagella and toxins. Further phenotypic characterization of *rho* mutants additionally linked the loss of Rho to altered sporulation and ability to colonize the intestine in a murine model of infection, indicating a broader role for Rho-mediated transcription termination in *C*. *difficile* physiology.

## Results

### Motile suppressors arise from a non-motile *recV flg* OFF mutant

**Fig 1:**
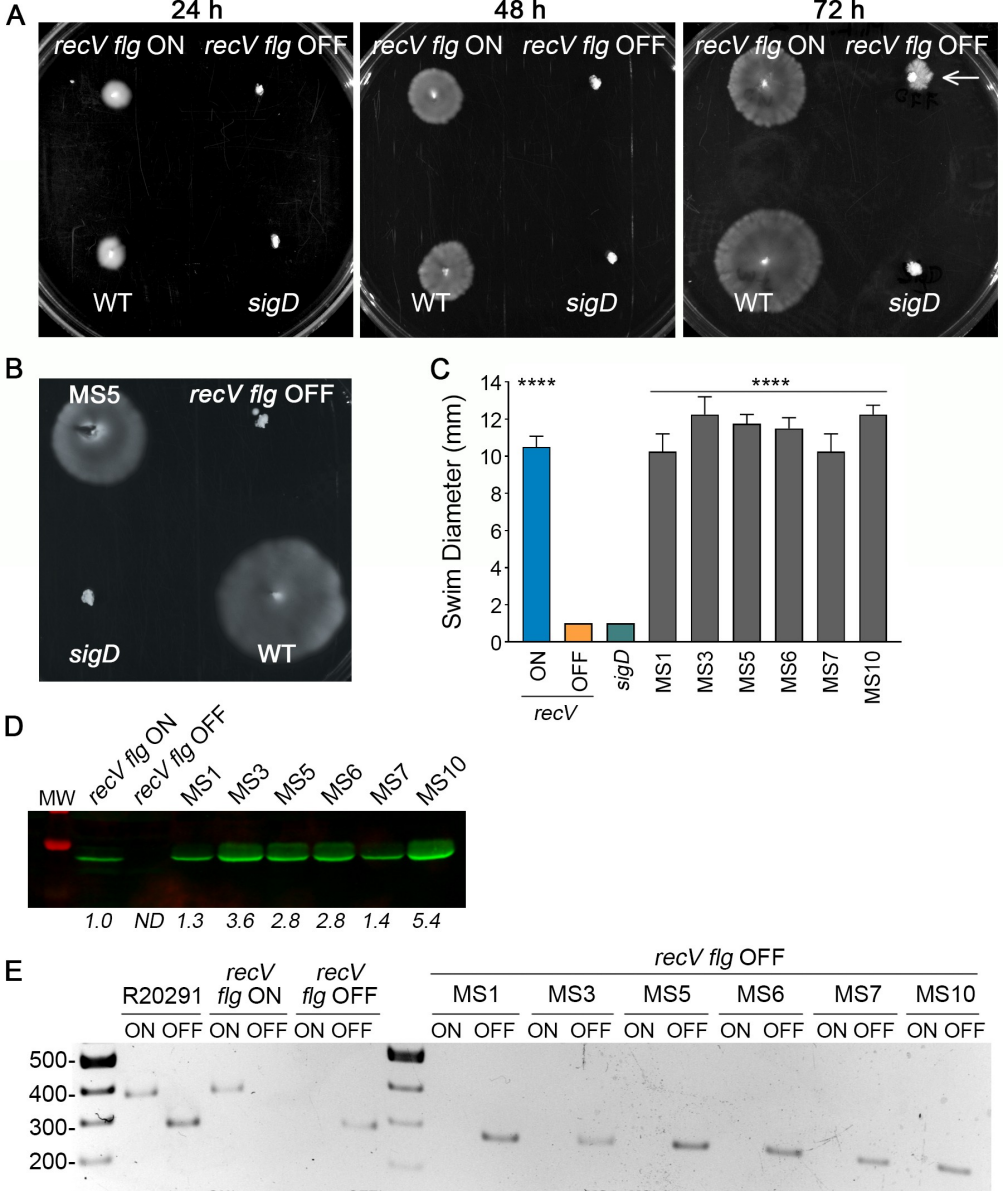
Motile suppressor mutants arise from a non-motile *recV flg* OFF strain. (A) Swimming motility in soft agar medium of *C*. *difficile* R20291 (WT), *recV flg* ON (RT1702), *recV flg* OFF (RT1693), and *sigD-*null non-motile control. Images were taken of representative plates after 24, 48 and 72 h. The arrow on the 72 h image points to a motile flare arising from *recV flg* OFF. (B) Swimming motility in soft agar medium of *C*. *difficile* R20291 (WT), *recV flg* OFF (RT1694), MS5, and *sigD*-null non-motile control. Image of a representative plate at 48 h. (C) Quantification of swimming motility of 6 motile suppressors (MS) selected for further characterization. Swim diameter was measured after 48 h. The means and standard deviation from 4 biological replicates are shown. ****p<0.0001 by one-way ANOVA and Dunnett’s post-test compared to *recV flg* OFF. (D) Immunoblot detection of FliC in *recV flg* ON and OFF and select MS. Shown is a representative image of three independent experiments. Numbers represent quantification of band intensity expressed as fold-change relative to *recV flg* ON. ND–non-detectable. (E) Orientation-specific PCR for flagellar switch in six motile suppressors, WT, and *recV flg* ON and OFF controls. Band sizes– 280bp (OFF) or 375bp (ON). Shown is a representative image of three independent experiments.

In *C*. *difficile* strain R20291 (ribotype 027), the DNA recombinase RecV is required for flagellar switch inversion, and loss of *recV* leads to phenotypically-locked bacteria [33]. These *recV* locked ON (*recV flg* ON, RT1702) and locked OFF (*recV flg* OFF, RT1693) strains are phenotypically distinct. As previously shown, *recV flg* ON bacteria are motile in soft agar, while *recV flg* OFF bacteria typically remain non-motile even after 72 hours (Fig 1A); in a subset of experiments, we observed spreading of the *recV flg* OFF mutant after prolonged incubation in soft agar (≥72 hours) (Fig 1A, right panel) [33]. In contrast to the uniform motility exhibited by WT and *recV flg* ON bacteria, these ‘motile flares’ were irregular and asymmetrical. The same phenomenon was observed for an independent *recV flg* OFF mutant, RT1694, which differs from RT1693 in the orientation of the invertible sequence upstream of *cwpV*, another phase variable locus (S1 Fig) [33, 48]. To recover the motile bacteria, which we term motile suppressors (MS) hereafter, we collected growth from the outer edge of the motile flares and subcultured on BHIS agar to obtain isolated colonies. When inoculated into soft agar, these MS showed motility similar to that of WT bacteria; an example is shown in Fig 1B. A total of 14 MS isolated from RT1693 and RT1694 were assayed for swimming motility in soft agar. All 14 MS regained swimming motility to the level of the *recV flg* ON strain, while the *recV flg* OFF and non-motile *sigD*-null control remained non-motile (S2 Fig); the results for 6 representative MS are shown in Fig 1C. Consistent with these phenotypes, production of the flagellin FliC was restored in these 6 representative MS, with FliC levels equivalent to or higher than *recV flg* ON (Fig 1D). FliC was undetectable in the *recV flg* OFF parental strain.

This observation suggested that the *recV flg* OFF mutant is capable of recovering motility. One possible explanation is that a recombinase other than *recV* catalyzed inversion of the flagellar switch. To test this possibility, we used orientation-specific polymerase chain reaction (OS-PCR), which employs primer pairs that specifically amplify one orientation of an invertible sequence or the other (S1 Table) [33]. Wild-type *C*. *difficile* R20291 grown in BHIS broth consists of a heterogeneous population. Accordingly, both OFF and ON flagellar switch orientations were detected by OS-PCR (Fig 1E). As determined previously, the *recV flg* OFF and *recV flg* ON strains yielded 280bp and 375bp product sizes, respectively, which correspond to the known flagellar switch orientations in these mutants [33]. The 14 MS contained the flagellar switch exclusively in the OFF orientation, with a representative 6 MS shown in Fig 1D. These results indicate that flagellar switch inversion did not occur, and the MS retain the *flg* OFF genotype despite their motile phenotype (Fig 1B). We also sequenced the promoter region and 5’ UTR of the *flgB* operon and did not find any mutations in the MS compared to the parental strains. Together, these results suggest that additional mechanisms are involved in inhibiting motility in *C*. *difficile* when the flagellar switch is in the OFF orientation.

### Identification of Rho as a negative regulator of flagellar motility

Soft agar swimming motility assays provide a strong selective pressure for bacterial self-propulsion to enable access to nutrients as they become depleted locally. The results above suggest that extragenic mutations arose in the MS that alleviated the negative effect of the *flg* OFF genotype on swimming motility in this assay. We postulated that a suppressor mutation occurred in a gene(s) involved in inhibiting flagellar gene expression in *recV flg* OFF bacteria. We thus performed whole genome sequencing of seven motile suppressors (MS 1–7) to identify single nucleotide polymorphisms (SNPs) compared to the R20291 wild type reference genome (detailed in S2 Table, S1 Data). Compared to the *recV flg* OFF strain, five of seven MS carried SNPs upstream of CDR20291_1465, which encodes a putative Mn^2+^-containing catalase. The same five MS (MS 2–6) also carried SNPs in the region between CDR20291_1414 and 1415, which are convergently transcribed and encode the putative acetolactate synthase subunit IlvB and a phage-associated integrase, respectively. Additional SNPs appeared within the inverted repeats of switches upstream of *cwpV* and the *flgB* operon, in accordance with these strains containing inversions in the *cwpV* and *flg* switches relative to the R20291 reference genome. Similarly, SNPs were also present in the inverted repeats flanking the invertible sequence upstream of CDR20291_0685, which was previously shown to be heterogeneous compared to the R20291 reference [44, 45].

**Fig 2:**
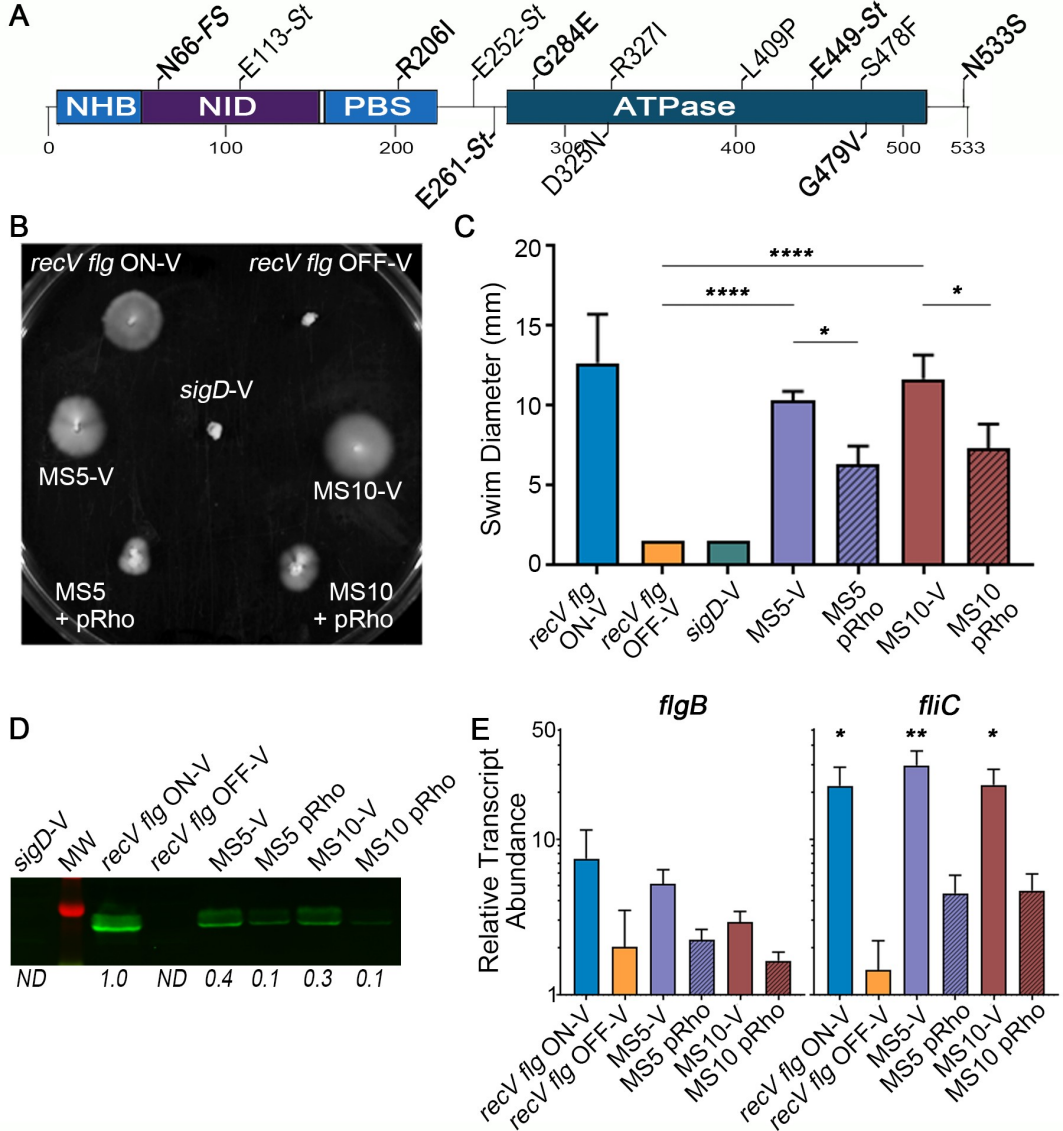
Rho is a negative regulator of flagellar motility. (A) Diagram of Rho factor domains and single nucleotide polymorphisms (SNPs) identified in the motile suppressors (MS). NHB–N-terminal helix bundle, NID–N-terminal insertion domain, PBS–primary binding site, FS–frameshift, St–stop codon. N66-FS results in a stop codon at residue 73. (B) Swimming motility in soft agar medium of motile suppressor 5 (MS5; N66-FS) and 10 (MS10; E113-St) expressing wild-type *rho* (pRho) or bearing vector at 48 hours. Controls included *recV flg* ON, *recV flg* OFF and non-motile *sigD* mutant carrying vector. (C) Quantification of swimming motility after 48 h of strains in (B). The means and standard deviation from 3 independent experiments are shown. ****p<0.0001, *p<0.05 by one-way ANOVA and Tukey’s post-test. (D) Immunoblot detection of FliC by strains in (B, C). Shown is a representative image of three independent experiments. Numbers represent quantification of band intensity expressed as the fold change compared to *recV flg* ON for the image shown. ND–non-detectable. (E) Relative transcript abundance of *flgB* and *fliC* measured by qRT-PCR. The means and standard deviation from 4 biological replicates are shown. **p<0.01, *p<0.05 by one-way ANOVA and Dunnett’s post-test compared to *recV flg* OFF.

A single locus, CDR20291_3324, contained SNPs in MS 1–7 compared to the allele in R20291 (Table 1, S1 Data); SNPs in this gene did not appear in either *recV flg* OFF parent. These SNPs showed a high frequency (89% or greater) in the sequencing reads. In lieu of whole genome sequencing, we Sanger sequenced CDR20291_3324 in MS 8–14 and identified additional SNPs in all of them. CDR20291_3324 encodes Rho factor, a transcriptional terminator known to act at the 3’ ends of genes or operons as an alternative mechanism to the use of an intrinsic terminator [49]. Rho binds nascent transcripts at Rho utilization (rut) sites, cytosine-rich sequences with poor consensus [49]. Hexameric Rho then uses ATPase activity to translocate 5’ to 3’ along the RNA. Although the mechanism of Rho-dependent transcription termination is not fully understood, it is thought to occur when Rho reaches the RNA polymerase, e.g. at an RNAP pause site, and forces its movement on template DNA without nucleotide addition, leading to destabilization of the transcription complex and mRNA release [50]. The SNPs identified in *rho* mapped to different domains of the Rho protein, including an N-terminal insertion domain (NID), primary binding site (PBS), and the C-terminal ATPase domain (Fig 2A). Mutations included substitutions (e.g. R206I, G284E) as well as nonsense mutations resulting either from a SNP creating a stop codon (e.g. E113-Stop) or a frameshift (e.g. N66-FS, resulting in a stop codon at residue 73).

**Table 1:** Summary of single nucleotide polymorphisms in *recV flg* OFF motile suppressor mutants.

*recV flg* OFF Suppressor	Position on Chromosome^a^	Nucleotide				Locus Tag	Gene	Amino Acid Change
		Ref	Alt	Freq^b^	Cover^b^			
MS1	3955138	T	C	99.28	978	3324	*rho*	N533S
MS2	3955300	C	A	99.13	2295	3324	*rho*	G479V
MS3	3955885	C	T	97.66	2055	3324	*rho*	G284E
MS4	3955391	C	A	99.05	2114	3324	*rho*	E449-Stop
MS5	3956539	T	/	89.83	2056	3324	*rho*	N66-FS
MS6	3955955	C	A	99.22	2055	3324	*rho*	E261-Stop
MS7	3956119	C	A	99.02	2447	3324	*rho*	R206I
MS8	1433	C	T	N/A	N/A	3324	*rho*	S478F
MS9	1226	T	C	N/A	N/A	3324	*rho*	L409P
MS10	337	G	T	N/A	N/A	3324	*rho*	E113-Stop
MS11	1598	A	G	N/A	N/A	3324	*rho*	N533S
MS12	980	G	T	N/A	N/A	3324	*rho*	R327I
MS13	973	G	A	N/A	N/A	3324	*rho*	D325N
MS14	754	G	A	N/A	N/A	3324	*rho*	E252-Stop

^a^ Note that *rho* is encoded on the complementary strand, and reference and alternate (Ref/Alt) nucleotides are designated based on chromosome position for MS#1–7 and gene position, i.e. sense orientation, for MS#8–14.

^b^ Frequency of the alternate nucleotide and coverage of reads at each of the indicated positions.

Rho is essential for viability in gram-negative bacteria but dispensable in gram-positive bacteria studied to date, such as *Bacillus subtilis* and *Staphylococcus aureus* [51, 52]. Based on a transposon mutagenesis screen, Rho is not essential for viability in *C*. *difficile* [53]. However, loss of Rho can cause growth defects potentially due to inappropriate, pervasive read-through transcription [54–56]. Therefore, we assessed the growth of the 14 motile suppressors in BHIS broth. All motile suppressors reached a lower final optical density (OD_600_) compared to *recV flg* OFF (S3 Fig). While doubling times during exponential growth for the *recV flg* ON and OFF strains were 56.5 ± 6.0 and 63.6 ± 3.5, respectively, growth rates for the MS ranged from 71.8 to 129.9 minutes, and 10 of the MS showed significant attenuation of doubling time (Table 2).

**Table 2:** Doubling times in rich medium.

Strain	Doubling Time (min)^a^^,^ ^b^
*recV flg* ON	56.5 ± 6.0
*recV flg* OFF	63.6 ± 3.5
MS1	75.7 ± 6.6
MS2	**129.9 ± 12.2**
MS3	**98.0 ± 10.5**
MS4	**122.2 ± 10.5**
MS5	**123.1 ± 27.8**
MS6	**126.1 ± 27.8**
MS7	75.9 ± 4.6
MS8	71.8 ± 5.5
MS9	**98.2 ± 3.5**
MS10	**91.0 ± 3.3**
MS11	76.0 ± 4.5
MS12	**103.5 ± 3.4**
MS13	**85.1 ± 1.5**
MS14	**97.0 ± 4.7**

^a^ Means and standard deviations for data from two experiments each with 3 biological replicates (n = 6).

^b^ Bold indicates p < 0.05 by one-way ANOVA and Dunnett’s post-test compared to *recV flg* OFF.

Because the *rho* mutations restored motility to the *recV flg* OFF mutants, we next tested the effect of expressing the wild-type *rho* allele in *rho*-null strains. We were unsuccessful in generating an in-frame deletion of *rho*, likely because of the associated growth defects. In lieu of a targeted mutant, we utilized MS5 and MS10, which contain stop codons early in the *rho* coding sequence: N66-FS (stop codon at position 73) and E113-St, respectively (Fig 2A). A plasmid carrying wild-type *rho* under control of an inducible promoter, pRho, was introduced into these MS, and the resulting strains were assayed for swimming motility. The vector control strains showed the expected swimming motility behaviors after 48 hours (Fig 2B and 2C). Expression of *rho* in MS5 and MS10 significantly inhibited swimming motility compared to the respective vector controls, effectively complementing the effects of the SNPs in *rho*. Consistent with these phenotypes, expression of *rho* led to 3- to 4-fold decreases in FliC levels in MS5 and MS10 compared to vector controls (Fig 2D). To examine whether changes in protein production and swimming were due to changes in flagellar gene transcription, we used quantitative reverse transcriptase PCR (qRT-PCR) to measure the transcript abundance of *flgB*, the first gene of the operon controlled by the flagellar switch, and *fliC*, which encodes flagellin and is regulated by SigD [22, 25]. The *flgB* transcript abundance was higher in MS5 and MS10 than *recV flg* OFF, though the differences were not significant (Fig 2E, left). Both MS5 and MS10 had significantly increased *fliC* transcript levels compared to *recV flg* OFF (Fig 2E, right). Providing *rho in trans* reduced *flgB* and *fliC* transcript abundance compared to the vector control. Notably, expression of *rho* also corrected the growth defect of MS5 and MS10 to the level of *recV flg* OFF parent with vector (S4 Fig). Together these results indicate that Rho regulates flagellum production and swimming motility in *C*. *difficile* by directly or indirectly inhibiting transcription of flagellar genes.

### Rho inhibits heterogeneous flagellar gene expression

**Fig 3:**
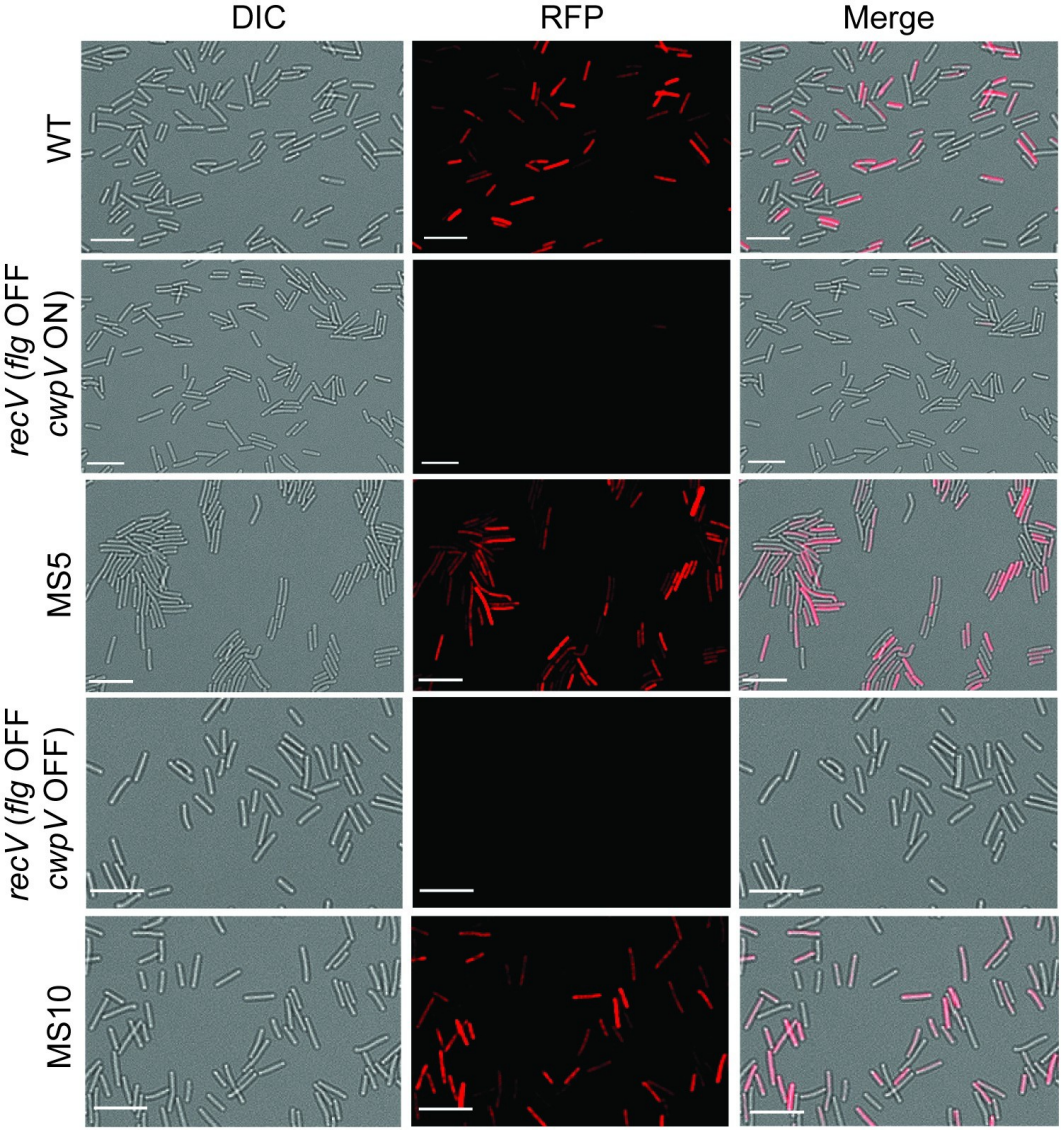
Rho inhibits heterogeneous flagellar gene expression in a *recV flg* OFF background. Micrographs of *C*. *difficile* R20291 (WT), MS5 and its *recV flg* OFF *cwpV* ON parent (RT1694), and MS10 and its *recV flg* OFF *cwpV* OFF parent (RT1693) transformed with the pP_*flgM*_::*mCherryOpt* reporter plasmid. Representative images for three independent experiments. Channels used are indicated for each column. White bars = 10 microns.

Analysis of flagellar gene expression by qRT-PCR reflects the transcript abundance averaged across the bacterial population. Yet, phase variation generates a heterogeneous population of bacteria, some of which express flagellar genes, and some of which do not. To analyze the effects of Rho on flagellar gene expression at the single cell level, we used fluorescence microscopy with a red fluorescence protein (mCherryOpt) reporter gene under the control of the *flgM* promoter (P_*flgM*_), which is a SigD-dependent promoter in the late stage flagellar operon [22, 23]. Expression of mCherryOpt from the *flgM* promoter is thus an indirect indication of the flagellar switch orientation. As previously observed, a population of wild-type *C*. *difficile* R20291 exhibited heterogeneity in fluorescence, with the majority of cells expressing mCherryOpt (Fig 3) [33]. In contrast, virtually no red cells were detectable for *recV flg* OFF *C*. *difficile*. Heterogeneous fluorescence was restored in MS5 and MS10 carrying P_*flgM*_::mCherryOpt (Fig 3). Thus, Rho is necessary for suppression of flagellar gene expression in *recV flg* OFF bacteria.

### Rho negatively impacts toxin production

**Fig 4:**
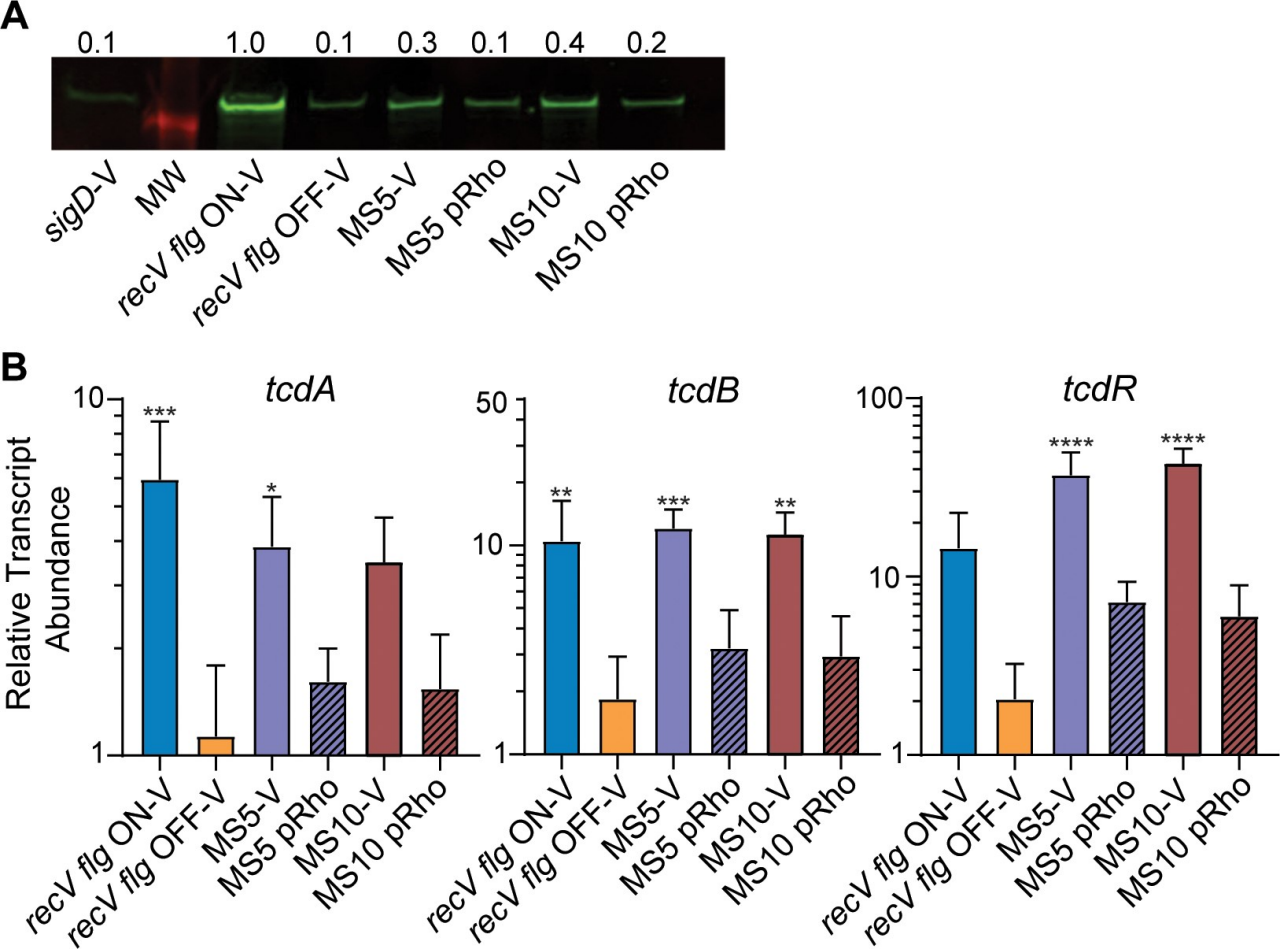
Mutations in *rho* impact toxin production. (A) Immunoblot detection of TcdA by MS5 and MS10 bearing pRho for expression of wild-type *rho* or bearing vector. Controls included *recV flg* ON, *recV flg* OFF and *sigD* mutant carrying vector. Shown is a representative image for three independent experiments. Numbers represent quantification of band intensity expressed as the fold change compared to *recV flg* ON for the image shown. (B) Relative transcript abundance of *tcdA*, *tcdB*, and *tcdR* measured by qRT-PCR. The means and standard deviation from 3 to 5 biological replicates per strain are shown. ****p<0.0001, ***p<0.001, **p<0.01, *p<0.05 by one-way ANOVA and Dunnett’s post-test compared to *recV flg* OFF.

The alternative sigma factor SigD, encoded in the *flgB* operon, positively regulates the expression of *tcdR*, *tcdA*, and *tcdB* [23, 27]. We therefore predicted that inhibition of *flgB* operon transcription by Rho would concomitantly inhibit toxin gene expression. We evaluated TcdA production in MS5 and MS10 carrying vector or pRho by immunoblot. MS5 and MS10 showed a 3- to 4-fold increase in TcdA production, respectively, compared to the *recV flg* OFF parent (Fig 4A). Expression of wild-type *rho* in MS5 and MS10 decreased TcdA levels, resulting in TcdA levels comparable to *recV flg* OFF bacteria. To determine whether changes in protein levels correlate with changes in transcript abundance, we examined expression of *tcdR*, *tcdA*, and *tcdB* by qRT-PCR. As observed previously, *tcdA* and *tcdB* transcript abundance was significantly higher in *recV flg* ON than *recV flg* OFF bacteria; *tcdR* was similarly altered, though the difference was not statistically significant (Fig 4B) [33]. Consistent with the negative impact of Rho on flagellar gene expression, *tcdA*, *tcdB*, and *tcdR* transcript levels were higher in MS5 and MS10 than in the *recV flg* OFF parent (Fig 4B). Providing *rho in trans* decreased *tcdA*, *tcdB*, and *tcdR* transcript abundances of MS5 and MS10 to the parental *recV flg* OFF levels. Therefore, in addition to inhibiting motility and growth, Rho negatively affects toxin gene expression and production. This effect is likely mediated through SigD encoded in the *flgB* operon [23, 27].

### Mutant *rho* alleles confer dominant negative motility phenotypes

**Fig 5:**
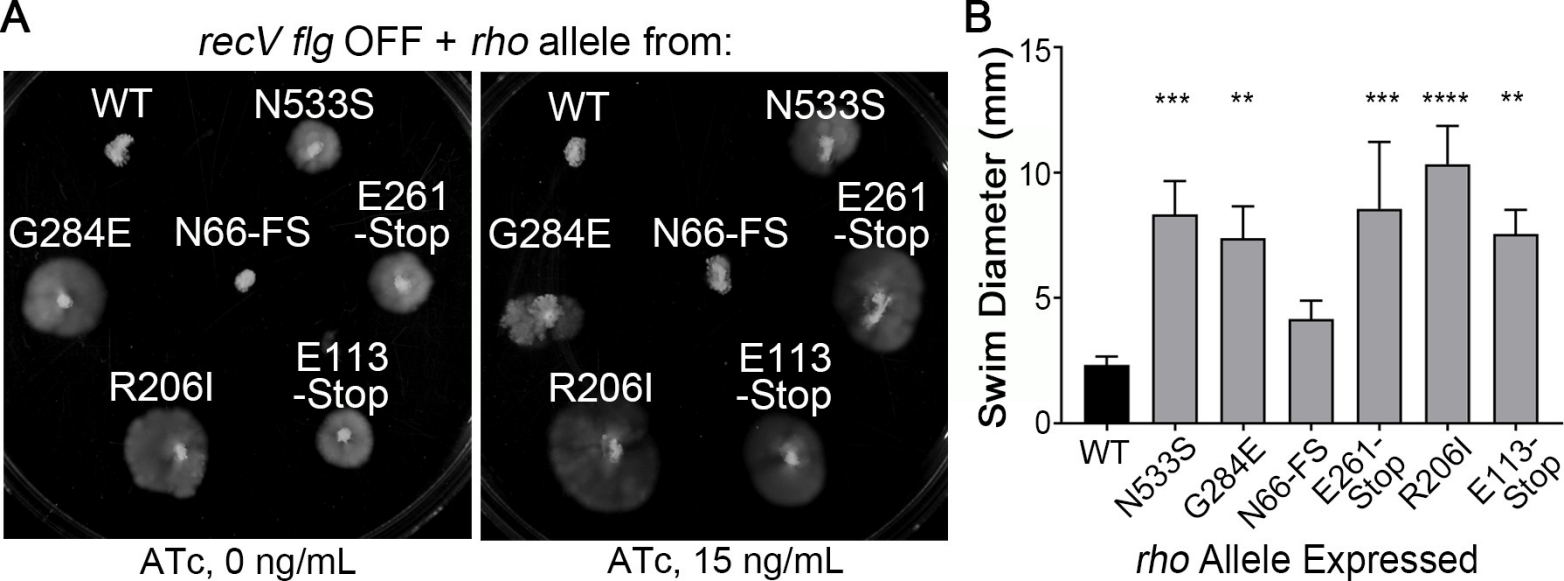
Mutant *rho* alleles confer dominant negative motility phenotypes. (A) Swimming motility in soft agar medium after 72 hours for the *recV flg* OFF (RT1693) expressing *rho* alleles encoding the indicated Rho proteins. Labels correspond to SNPs in *rho* (Fig 2A), and WT corresponds to the wild-type *rho* allele. Dominant negative effects are present regardless of anhydrotetracycline (ATc) induction suggesting leaky expression from the P_*tet*_ promoter. (B) Quantification of swim diameter from soft agar plates containing 15ng/mL ATc after 72 hours. The means and standard deviation of three biological replicates are shown. ****p<0.0001, ***p<0.001, **p<0.01 by one-way ANOVA and Dunnett’s post-test compared to *recV flg* OFF.

Rho factor functions as a homohexamer [57]. We hypothesized that overexpression of mutant *rho* alleles could result in incorporation of aberrant subunits into the hexamer, interfering with Rho function. To test for this dominant negative effect, we introduced mutant *rho* alleles from six different MS into the *recV flg* OFF strain, with the expectation that incorporation of non-functional Rho monomers would prevent inhibition of swimming motility by the wild-type, chromosomally-encoded Rho. The wild-type *rho* allele was also introduced as a control. To achieve overexpression of the cloned alleles, the genes were placed under the control of an anhydrotetracycline (ATc)-inducible promoter in the multi-copy plasmid pRPF185. Strains bearing these expression plasmids were assayed for swimming motility in soft agar medium. Expression of five of the six of the mutant *rho* alleles restored swimming motility to varying extents, while the wild-type allele did not alter motility (Fig 5A and 5B). These results were obtained regardless of ATc induction, suggesting leaky expression from the P_*tet*_ promoter as previously reported [58, 59]. The only mutant allele that did not lead to a dominant negative motility phenotype was N66-FS derived from MS5 (Fig 2A). Because this *rho* allele contains a nonsense mutation resulting in an early stop codon at residue 73, the truncated gene product may be unstable or unable to incorporate into the Rho hexamer, further justifying the use of the MS5 strain as a *rho*-null mutant.

### Rho preferentially inhibits transcription of *flg* OFF mRNA

**Fig 6:**
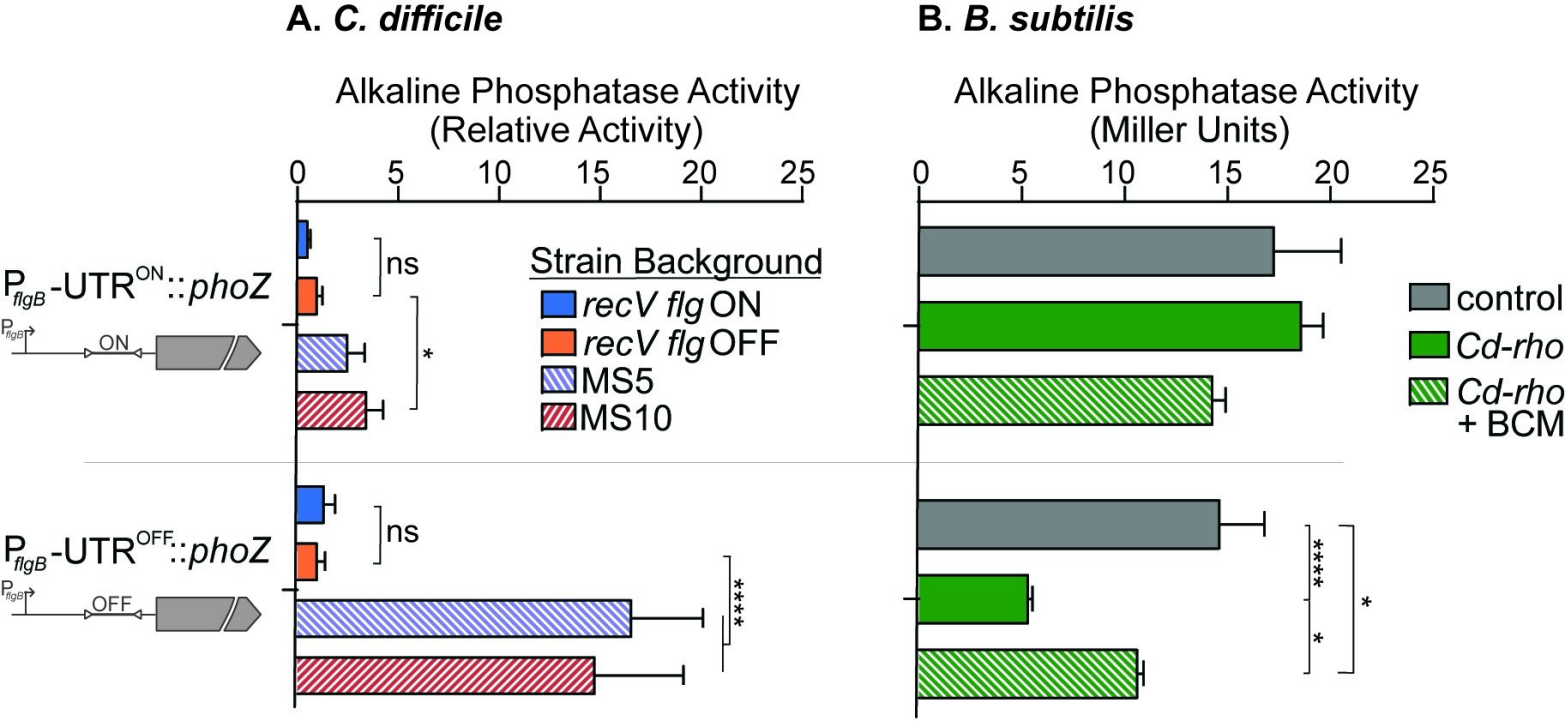
*C*. *difficile* Rho directly inhibits flagellar gene expression in *flg* OFF orientation. **(**A) Alkaline phosphatase (*phoZ*) reporter fusions to the *flgB* operon regulatory region, with the flagellar switch in either the ON or OFF orientation, were introduced into the *recV flg* ON (RT1702), *recV flg* OFF (RT1693), MS5 and MS10 strains. Alkaline phosphatase activity was normalized to the *recV flg* OFF values. The means and standard deviation of 5 biological replicates are shown. ****p<0.0001 by two-way ANOVA and Tukey’s post-test. (B) The *C*. *difficile rho* gene was introduced into *Bacillus subtilis* bearing the *phoZ* fusions to the *flgB* regulatory region with the flagellar switch in either the ON or OFF orientation. *C*. *difficile rho* (*Cd-rho*) was introduced, and alkaline phosphatase activity was assayed. Control–*B*. *subtilis* reporter without *Cd-rho*; BCM–bicyclomycin, 50 μg/mL. The means and standard deviation of three biological replicates are shown. ****p<0.0001, *p<0.05 by two-way ANOVA with Sidak’s post-test comparing strains with the same reporter construct.

Our prior work suggests that transcription termination of *flg* OFF bacteria is mediated by a *trans*-acting factor specific to *C*. *difficile* [33]. This role could be fulfilled by Rho. While Rho typically terminates transcription of genes and operons 3’ of coding sequences, recent studies have shown that Rho can also terminate transcription in some 5’ leader regions in gram-negative bacteria [60–63]. This mechanism would have a regulatory effect on the downstream gene(s). We therefore hypothesized that Rho is this *trans*-acting factor affecting transcription of the *flgB* operon, preferentially inhibiting transcription in *flgB* transcripts containing the flagellar switch in the OFF orientation. To test this hypothesis, we transcriptionally fused a *phoZ* reporter gene to the *flgB* coding sequence and the upstream regulatory region of the *flgB* operon. This 1045 bp region includes the σA-dependent promoter and the 498 bp 5’ untranslated region containing with the flagellar switch in either the ON or OFF orientation: P_*flgB*_*-*UTR^ON^::*phoZ* and P_*flgB*_*-*UTR^OFF^::*phoZ* respectively. Promoterless (::*phoZ*) and promoter-only constructs (P_*flgB*_::*phoZ*) were included as controls. These plasmid-borne reporters were introduced into *recV flg* ON and OFF strains, which encode wild-type Rho, and MS5 and MS10, which contain mutant *rho* alleles, and alkaline phosphatase activity was assayed [64]. As anticipated, the no-promoter control lacked activity in all strains, and no differences were observed for activity of the promoter only reporter suggesting that Rho does not regulate transcription at the level of promoter (S5 Fig). For the P_*flgB*_*-*UTR^ON^::*phoZ* reporter, activity was modestly (~3-fold) higher in MS5 and MS10 compared to the *recV flg* OFF parent (Fig 6A). In comparison, activity was ~15-fold higher in MS5 and MS10 compared to *recV flg* OFF for the P_*flgB*_*-*UTR^OFF^::*phoZ* reporter. Therefore, mutation of *rho* had a greater effect on flagellar gene transcription for bacteria with the flagellar switch in the OFF orientation, suggesting that Rho preferentially inhibits transcription of the *flg* OFF transcript.

The results of alkaline phosphatase assays in *C*. *difficile* imply that Rho negatively regulates *flgB* operon transcription, but do not distinguish between a direct and indirect mechanism of regulation. Rho could directly act on the *flgB* UTR to terminate transcription from *flgB* OFF mRNA, or Rho could impact the production of another protein involved in *flgB* regulation. We previously showed that, whereas the P_*flgB*_*-*UTR^ON^::*phoZ* reporter resulted in significantly higher activity than the P_*flgB*_*-*UTR^OFF^::*phoZ* reporter in *C*. *difficile*, the difference was lost when these reporters were assayed in *B*. *subtilis* [33]. These results indicate that *B*. *subtilis* does not encode the factor that mediates regulation. We postulated that if Rho directly terminates transcription within the *flgB* 5’ UTR, introducing *C*. *difficile rho* (*Cd-rho*) into *B*. *subtilis* strains carrying the P_*flgB*_*-*UTR^ON^::*phoZ* and P_*flgB*_*-*UTR^OFF^::*phoZ* reporters would restore the regulation seen in *C*. *difficile*. To test this idea, the wild-type *Cd-rho* allele was introduced into the previously constructed *B*. *subtilis* reporter strains [33], and alkaline phosphatase activity was assayed. As seen previously, reporter activity was the same in *B*. *subtilis* with P_*flgB*_*-*UTR^ON^::*phoZ* and P_*flgB*_*-*UTR^OFF^::*phoZ* (Fig 6B). Expression of *Cd-rho* resulted in decreased activity only in *B*. *subtilis* with the P_*flgB*_*-*UTR^OFF^::*phoZ* reporter. Finally, the addition of bicyclomycin, a specific inhibitor of Rho ATPase activity [65], abrogated this effect. These data indicate that Rho, and not another *C*. *difficile* protein, is the *trans*-acting factor which directly inhibits flagellar gene expression, and that Rho selectively prevents transcription in *flg* OFF bacteria.

### Rho is important for early colonization in a mouse model of infection and efficient sporulation

*In vitro*, Rho affects several phenotypes including growth, motility, and toxin production. Because these characteristics are important during CDI, we analyzed the effect of a *rho* mutation in a mouse model of infection. MS5 and MS10 were derived from two *recV* mutant strains that differ in *cwpV* status–MS5 was derived from RT1694 (*cwpV* ON) while MS10 was derived from RT1693 (*cwpV* OFF) [48]. The role of *cwpV in vivo* has not been previously reported, so both *recV flg* OFF parental strains were tested. RecV is required for site-specific recombination of multiple invertible sequences, not only the flagellar and *cwpV* switches [44, 45, 48]. To ensure appropriate attribution of phenotypes, we confirmed that the parental strains and motile suppressors are isogenic for the other sequences and only differ in the *cwpV* switch (S6A Fig). Male and female C57BL/6 mice were treated with antibiotics to render them susceptible to *C*. *difficile* and then inoculated by oral gavage with 10^5^ spores of wild-type R20291, MS5, MS10, and their respective parent strains.

**Fig 7:**
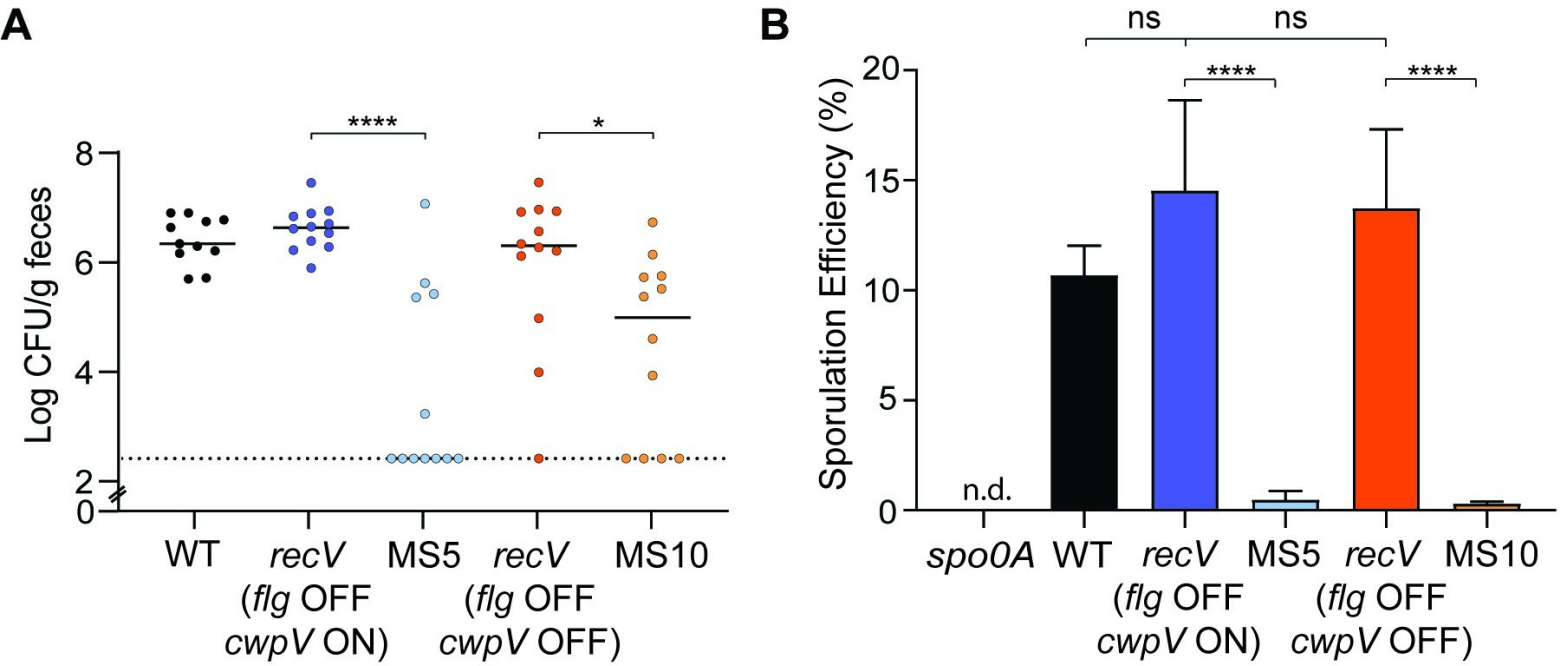
Rho is important for early colonization in a mouse model of infection and efficient sporulation. (A) Antibiotic-treated male and female C57BL6 mice were inoculated with 10^5^ spores of the indicated *C*. *difficile* strain. CFU in feces collected at 24 hours post inoculation were enumerated. Data are combined from two independent infection studies that each included 3 male and 3 female mice for 12 total mice per strain. Symbols indicate CFU from individual animals, and bars indicate the medians. ****p<0.0001, *p<0.05 by Kruskal-Wallis test with Dunn’s post-test comparing motile suppressors to their parent strains: RT1694 (*recV flg* OFF *cwpV* ON) and MS5, RT1693 (*recV flg* OFF *cwpV* OFF) and MS10. Dotted line represents a limit of detection. (B) Sporulation efficiency was evaluated by ethanol resistance and calculated as the total number of spores divided by the total number of viable cells (spores plus vegetative). A sporulation-deficient *spo0A* mutant was included as a control. The means and standard deviation of four biological replicates are shown. ****p<0.0001 by one-way ANOVA with Tukey’s post-test comparing all strains. n.d.–non-detectable.

As observed previously, wild-type R20291 colonized the mice within 1 day post-inoculation (reaching more than 10^6^ CFU/g feces), maintained this level of colonization for 1–3 days, then was gradually cleared typically between days 3 and 7 post-inoculation (S6B Fig). A similar, but not identical pattern of colonization was seen for both *recV flg* OFF strains, suggesting that *cwpV* expression does not consistently impact colonization in this model. For MS5 and MS10, the bacterial burden in feces was significantly lower on day 1 post-inoculation compared to the respective *recV flg* OFF parents and wild-type R20291 (Fig 7A). About 50% of the animals inoculated with MS5 or MS10 had undetectable levels of *C*. *difficile* in their feces; most of the remaining animals showed intermediate or parental levels of colonization. Interestingly, colonization of both MS5 and MS10 recovered to parental levels starting at day 2 post infection and were cleared within a similar time frame (S6B Fig), suggesting that Rho is important for initial colonization in a mouse model of infection. Notably, although the *recV flg* ON, MS5, and MS10 strains differ in toxin production compared to the *recV flg* OFF strain *in vitro*, we did not observe significant differences in weight loss or diarrheal symptoms between the groups of infected animals. These results may be attributable to the subclinical colitis caused by R20291 in this animal model, as previously reported [66–68]. It is also possible that other regulators of toxin gene expression, such as CodY and CcpA, unlink co-expression of the toxin and flagellar genes [69–72].

Sporulation and germination are important for colonization of the mouse model [73]. Because both MS5 and MS10 were attenuated for colonization on day 1 of infection, we considered that this difference is attributable to a germination and/or sporulation defect. We assessed sporulation and spore viability by enumerating ethanol resistant spores as a percentage of total cells (spore plus vegetative) [74]. While the sporulation efficiency for the wild type and both *recV flg* OFF strains was between 10 and 15%, sporulation efficiency was <1% for both motile suppressors (Fig 7B). Our data implicate Rho as an important factor that positively regulates sporulation, which may contribute to the colonization defect observed in the mouse model. It is possible that additional SNPs in the motile suppressors also contribute to the sporulation defect.

To examine germination, purified spores of wild-type R20291, both *recV flg* OFF strains, MS5, and MS10 were assayed in buffer supplemented with the spore germinant 10 mM taurocholic acid (TA) as previously described [75]. In the absence of TA, no germination was detected. In the presence of TA, all strains germinated to the same level, indicating that Rho does not influence germination rate (S7 Fig).

## Discussion

In this study, we identified Rho as a trans-acting factor that controls phase variation of flagella and toxins *in vitro*. The regulation exerted by Rho contributes to the ability of a population of *C*. *difficile* to continually maintain motile, toxin-producing *flg* ON cells as well as non-motile, atoxigenic *flg* OFF cells. Comparative transcriptional analyses using *C*. *difficile* as well as *B*. *subtilis* as a heterologous system support that Rho inhibits flagellar gene transcription selectively in *flg* OFF bacteria. These results implicate Rho as an important regulatory component mediating phase variation of flagella, and by extension toxins, and reveal a new role for Rho-mediated transcription termination in regulation of gene expression.

All of the 14 motile suppressors (MS) contained nucleotide polymorphisms in Rho conferring a missense or nonsense mutation that presumably abrogated Rho function. Five of six *rho* alleles, which correlated with a range of growth defects in the respective MS, led to a dominant negative effect and relieved inhibition of motility in *flg* OFF bacteria when over expressed. In *E*. *coli*, a dominant negative effect resulted from less efficient binding of mRNA to the Rho secondary binding site and decreased translocation of Rho along the mRNA towards RNA polymerase [76]. In *C*. *difficile*, incorporation of mutant subunits into the Rho homohexamer could negatively affect mRNA binding, ATP processing and/or resulting helicase activity. These mechanisms are not mutually exclusive–mutations in different domains of Rho may affect Rho activity by different mechanisms while imparting the same effect on motility. We note that we were unable to generate an independent mutation in *rho* in a wild-type R20291 background, however the MS5 and MS10 strains with truncated Rho represent useful mutants for characterization of *rho*-null *C*. *difficile*. Further studies will determine how the mutant Rho proteins are altered in function and the mechanism by which mutant *rho* alleles interfere with function of wild-type Rho.

Rho suppressor mutants exhibit restored motility compared to the *flg* OFF bacteria from which they were derived. The increased motility of motile suppressors corresponds with increased expression of flagellar genes *flgB* and *fliC* and higher levels of the major flagellin FliC. Furthermore, Rho contributes to heterogeneity of flagellar expression at the single cell level as evidenced by differences in mCherry signal driven by the SigD-dependent *flgM* promoter. Unlike the *recV flg* OFF strain that lacks fluorescence, motile suppressors derived from *flg* OFF bacteria are mCherry positive and appear similar to a wild-type population. That loss of Rho in the MS resulted in heterogenous fluorescence intensity among individual cells indicates that another factor influences expression. A c-di-GMP riboswitch is encoded between the *flgB* transcriptional start site and the flagellar switch, and we speculate that fluorescence intensity reflects varying levels of c-di-GMP [77, 78].

**Fig 8:**
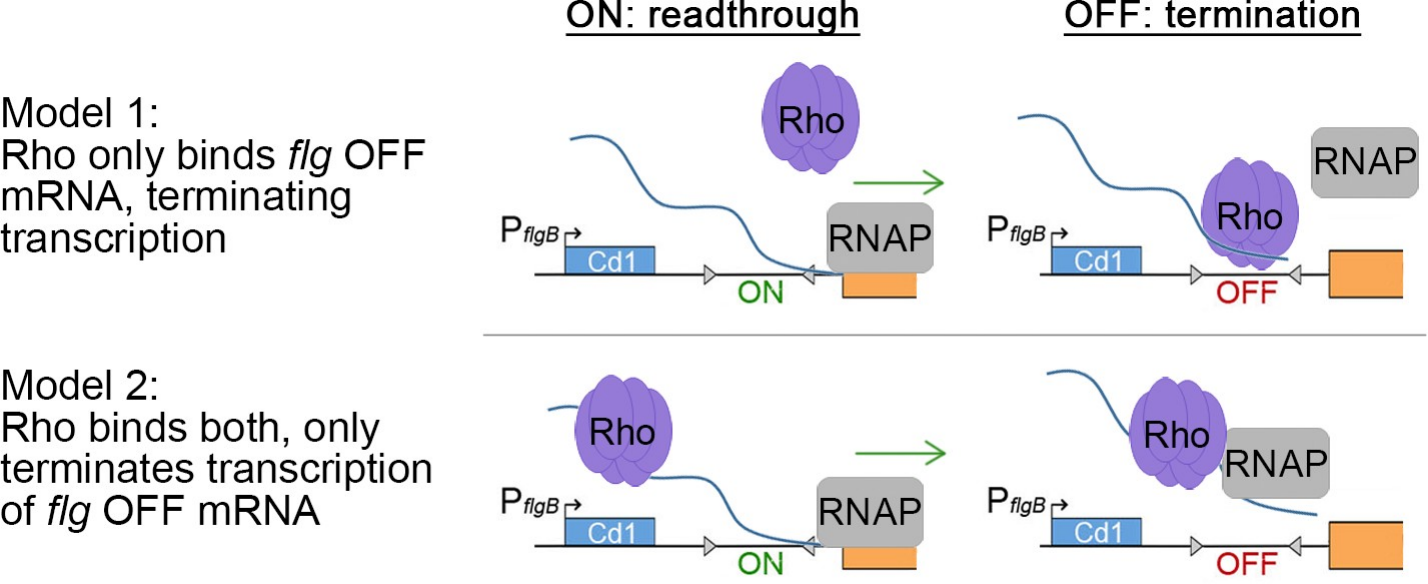
Proposed models of direct Rho binding and inhibition of transcription readthrough of *flg* OFF mRNA. Model 1: Rho distinguishes between *flg* ON and OFF mRNA by preferentially binding to *flg* OFF mRNA. This may be due to the presence of *rut* sequences that are absent in the *flg* ON. Model 2: Rho binds *flg* ON and OFF mRNAs equally, 5’ of the flagellar switch. Transcription termination may then be differentially influenced by the presence of an additional sequence required for termination (e.g. a RNA polymerase pause site) only in *flg* OFF mRNA.

Experiments with transcriptional *phoZ* fusions in *C*. *difficile* and *B*. *subtilis* indicate that Rho strongly inhibits transcription when the flagellar switch is in the OFF orientation. We propose two alternative models for direct control of phase variable expression of the *flgB* operon by Rho through the selective, premature termination of *flg* OFF transcripts (Fig 8). In model 1, Rho distinguishes between *flg* ON and OFF mRNA by preferentially binding to *flg* OFF mRNA due to the presence of *rut* sequences that are absent in the *flg* ON. In model 2, Rho binds *flg* ON and OFF mRNAs equally, 5’ of the flagellar switch. Transcription termination may then be differentially influenced by the presence of an additional sequence required for termination. For example, an RNA polymerase pause site may appear only in *flg* OFF mRNA [61]. In either model, Rho would selectively terminate *flgB* operon transcription and inhibit linked phenotypes in bacteria with the flagellar switch in the OFF orientation. More work is needed to distinguish between these two models. Using the RhoTermPredict algorithm [79], we were unable to identify any predicted *rut* sites within the *flgB* leader sequence of either *flg* ON or OFF sequences. RhoTermPredict is based on *E*. *coli*, *B*. *subtilis*, and *S*. *enterica* databases and searches for *rut* sites with regularly spaced C residues and C>G content followed by a putative RNA polymerase pause site [79]. However, the *C*. *difficile* genome has low G+C content (<30%) [80], and the *flgB* leader sequence has 23% G+C content. The *rut* site characteristics in *C*. *difficile* therefore may be different from those previously described in other bacteria, so the RhoTermPredict algorithm may be unsuitable for predicting *rut* sites in *C*. *difficile*.

There are several examples of Rho exerting regulation on 5’ leader sequences in gram-negative bacteria, where gene regulation by Rho is achieved by multiple mechanisms involving other proteins, small RNAs, and potentially yet unidentified factors. In *E*. *coli*, Rho seems to preferentially regulate expression of >250 genes with long 5’ UTRs [81]. In addition, the RNA-binding protein CsrA binds to the 5’ UTR of *pgaA* to prevent the formation of an RNA secondary structure that otherwise sequesters the *rut* site [82]. In *S*. *enterica* serovar Typhimurium, Rho binds within the leader sequences of three genes encoding Mg^2+^ transporters to control expression [60, 62, 83], and the small RNA ChiX inhibits expression of the *chiPQ* operon by inducing premature Rho-dependent termination [84]. Finally, most known *E*. *coli* riboswitches modulate gene expression by either translational regulation or Rho-dependent termination [60, 85]. However, riboswitches in *C*. *difficile*, including the c-di-GMP riboswitch upstream of the *flgB* operon, appear to act through Rho-independent mechanisms [77, 78]. To our knowledge, this is the first example of Rho-mediated transcription termination within a 5’ UTR that results in modulation of downstream gene expression in a gram-positive species.

In *E*. *coli*, Rho requires cofactors NusA and NusG to terminate transcription at many sites [86]. NusG and NusA are essential for growth of *C*. *difficile* R20291 [53], but the cofactor requirements for *C*. *difficile* Rho are currently unknown. Introduction of *C*. *difficile rho* into a heterologous host *B*. *subtilis* did not alter the ability of *C*. *difficile* Rho to terminate transcription of the *flg* OFF construct. These data suggest that either Rho is able to terminate flagellar transcription without additional cofactors, or it is able to use homologs of NusA, NusG, or other potential cofactors present in *B*. *subtilis*. Interestingly, while both *C*. *difficile* and *B*. *subtilis* encode Rho, only the *C*. *difficile* factor terminates *flg* OFF transcription. This difference could be caused by a difference in structure of these two proteins. Although many of the features of Rho are conserved across bacteria, in ~35% of species, including *C*. *difficile*, Rho contains an N-terminal insertion domain (NID) whose length and composition are not conserved among species [87]. In other bacterial species with an NID-containing Rho, the NID imparts diverse functions [88–90]. It is therefore possible that the insertion domain of *C*. *difficile* Rho confers the ability to terminate *flg* OFF transcription.

Mutations in *rho* negatively affect initial colonization in a mouse model of infection, resulting in a delay in colonization. We ruled out contributions from other phase-variable loci by ensuring that MS5 and MS10 are isogenic with the parental strains at these sites. The *rho* mutations likely have pleiotropic effects that impact colonization [91, 92], however the delayed colonization may be due in part to the defects in growth and sporulation of the motile suppressor mutants. Interestingly, a high-throughput screen in *C*. *difficile* R20291 did not identify *rho* as a gene required for sporulation [53]. How Rho affects growth and sporulation in *C*. *difficile* is unknown, but may arise from pervasive transcription, particularly loss of suppression of antisense transcription, or other potential consequences of loss of Rho [86, 93–95]. Further studies are needed to elucidate the effects of Rho on global transcription in *C*. *difficile* to determine the cause of the observed growth defects as well as other phenotypes affected by Rho.

## Materials and methods

### Growth and maintenance of bacterial strains

Strains and plasmids used in this study are listed in S3 Table. *C*. *difficile* was maintained in an anaerobic chamber (Coy Laboratories) in an atmosphere of 85% N_2_, 5% CO_2_, and 10% H_2_. *C*. *difficile* and *B*. *subtilis* were routinely cultivated in Brain Heart Infusion medium (Becton Dickinson) supplemented with 5% yeast extract (Becton Dickinson) (BHIS) at 37°C. Where indicated, bacteria were cultured in Tryptone Yeast (TY) broth. All *C*. *difficile* broth cultures were grown statically, with 10 μg/mL thiamphenicol (Tm) for plasmid maintenance as needed. *E*. *coli* DH5α and HB101(pRK24) were cultured under aerobic conditions at 37°C in Luria-Bertani (LB) broth. For selection of plasmids in *E*. *coli*, 100 μg/mL ampicillin (Amp) and/or 10 μg/mL chloramphenicol (Cm) was used, as indicated. Kanamycin (Kan) 100 μg/mL was used to select against *E*. *coli* after conjugations with *C*. *difficile*. Spectinomycin (Spec) 100 μg/mL was used to select for *B*. *subtilis* transformants containing *Cd-rho*.

### Soft agar swimming motility assay

Flagellum-dependent swimming motility was assayed in 0.5X BHIS-0.3% agar as previously described [25]. When appropriate, Tm was added for plasmid maintenance, and 10 ng/mL anhydrotetracycline (ATc) was added to induce gene expression. The diameter of motile growth was measured after 24, 48, and 72 hours. Three independent experiments were performed, each with six technical replicates. Images were taken using the G:BOX Chemi imaging system with the Upper White Light illuminator.

### Isolation and sequencing of motile suppressor mutants

The *recV flg* OFF mutants RT1693 and RT1694 (which contain the *cwpV* switch in the OFF or ON orientation, respectively) were grown in BHIS broth until OD_600_ of 1, then 1.5 μL were inoculated into 0.5X BHIS-0.3% agar motility medium and incubated at 37°C for 48–96 hours. Each plate included a non-motile *sigD* negative control (RT1566) and *recV flg* ON (RT1702) and *recV flg* OFF controls. Plates were examined for expansion of the *recV flg* OFF colonies, which appeared in a subset of plates. Bacteria were collected from the outer edge of motile growth and subcultured on BHIS agar.

Genomic DNA was extracted from seven motile isolates (RT1705 to RT1711 (MS 1–7), S3 Table) and the parental *recV flg* OFF strains (RT1693, RT1694) as previously described [96]. Genomic DNA of MS 1–7 was prepared using the KAPA HyperPrep Kit (Roche) and sequenced using an Illumina HiSeq 2500 Rapid Run platform with paired ends and 100X coverage by the UNC-CH High Throughput Genomic Sequencing Facility. The sequencing data is available on the National Center for Biotechnology Information (NCBI) Sequence Read Archive Database, accession number PRJNA630461. Sequencing reads were mapped to the reference *C*. *difficile* R20291 genome (Accession No. FN545816.1) using CLC Genomics Workbench v. 9 software (Qiagen), and nucleotide polymorphisms were identified using the fixed ploidy variant detector function with default parameters. Whole genome sequencing was not performed for MS 8–14. Instead, for MS 8–14 (RT1939-1945), the *rho* gene (CDR20291_3324) was amplified by PCR with primers R2307 and R2308, and the products were Sanger sequenced using primers R2307, R2308, R2366, and R2367. Primer sequences are provided in S1 Table. Nucleotide polymorphisms were identified by alignment with the wild-type sequence from R20291 using ClustalOmega [97].

### Determination of invertible switch orientation by orientation-specific PCR

*C*. *difficile* was cultured from glycerol stocks on BHIS agar for 24 hours at 37°C. A single colony was suspended in 20 μL of dH_2_O and heated at 100°C for 10 minutes. These lysates served as templates for PCR using primers that discriminate between each flagellar switch sequence orientation in R20291 (S1 Table). Primers R1614 and R857 were used to amplify the ON orientation of the flagellar switch, which corresponds to the published sequence of R20291. Primers R1615 and R857 were used to amplify the OFF orientation of the flagellar switch. Similarly, orientation-specific PCR was used to determine the orientations of the other invertible sequences using primers listed in S1 Table, which follow the naming pattern LOCUS_pubF and LOCUS_R for detection of the orientation in R20291 reference genome, and LOCUS_invF and LOCUS_R for the inverse orientations. Three independent experiments were done.

### Detection of FliC and TcdA by immunoblot

Western blots for TcdA and FliC production were performed as previously described [33, 34]. Cultures for TcdA immunoblotting were grown in TY broth overnight (~16 hours), diluted 1:50 in fresh TY broth, and grown until late stationary phase (OD_600_ of 1.8 to 2.0). Cultures for immunoblotting FliC were grown overnight (~16 hours) in BHIS broth. For complementation experiments, Tm was included in all growth media, and 10 ng/mL ATc was added to induce gene expression. For both FliC and TcdA detection, samples were normalized to an OD_600_ 1.0, and then cells were collected by centrifugation at 16,000 x g for 5 minutes (TcdA) or 2,000 x g for 10 minutes (FliC). Bacterial pellets were suspended in 1x SDS-PAGE sample buffer. The lysates were separated on a 12% SDS-polyacrylamide gel for FliC detection or on an 8% SDS-polyacrylamide gel for TcdA detection, then transferred to a nitrocellulose membrane (Bio-Rad). Membranes were stained with Ponceau S (Sigma) to assess equal loading and imaged using the G:Box Chemi imaging system. FliC was detected using α-FliC hamster sera (generous gift from Dr. Ghose-Paul) [34, 98] followed by goat anti-hamster IgG (H+L) secondary antibody conjugated to DyLight 800 (Novus Biologicals). TcdA was detected using mouse α-TcdA antibody (Novus Biologicals) followed by goat anti-mouse IgG secondary antibody conjugated to DyLight 800 4x PEG (Invitrogen). Blots were imaged using the Odyssey imaging system (LI-COR), and quantification was performed with Image Studio Software. All strains were assayed in at least three independent experiments.

### Growth curves

Overnight cultures were diluted 1:50 into BHIS medium including 10 μg/mL Tm and 10 ng/mL ATc as needed. Optical density (OD_600_) was measured every 30 minutes for 8 hours. Doubling times were calculated based on the change in optical density during exponential growth. Six biological replicates were assayed in two independent experiments.

### Quantitative reverse transcriptase-PCR

Overnight cultures were diluted in BHIS medium containing thiamphenicol as needed. Cells were grown to mid-exponential phase (OD_600_ 0.8–1) or stationary phase (OD_600_~1.5) for analysis of flagellum (*flgB*, *fliC*) and toxin (*tcdA*, *tcdB*, *tcdR*) gene expression, respectively. RNA was isolated as described previously [33, 96]. Briefly, cells were collected by centrifugation and stored in ethanol:acetone (1:1) at -80°C overnight. Cells were lysed by bead beating in cold TriSURE (Bioline). Nucleic acids were extracted with chloroform, precipitated from the aqueous phase with isopropanol, washed with ethanol, and suspended in RNase-free water. RNA was treated with TURBO DNase (Thermo Fisher) according to the manufacturer’s protocol. Synthesis of cDNA was done using the High-Capacity cDNA Reverse Transcription Kit (Applied Biosystems) and random hexamers according to the manufacturer’s instructions. No-reverse transcriptase controls were included in all experiments. Real-time PCR was performed using 10 ng of cDNA, a final primer concentration of 1 μM, and SYBR Green Real-Time qPCR reagents (Bioline). Relative transcript abundance was calculated using the ΔΔCt method, with *rpoC* as the control gene and the indicated reference condition/strain. Primers used are listed in S1 Table, with forward and reverse primers named according to the pattern *gene*-qF and–qR, respectively.

### Visualizing heterogeneity using fluorescent reporters

To visually examine population heterogeneity, we used a previously described protocol [99, 100]. Briefly, overnight cultures of strains containing the P_*flgM*_::*mCherryOpt* reporter were diluted 1:100 into BHIS-Tm. Bacteria were grown anaerobically at 37°C until OD_600_ ~0.5, 1 mL of culture was collected by centrifugation, and the remaining steps were performed aerobically. Cell pellets were washed with PBS, then suspended in 500 μl PBS and 120 μl 5x fixative (20 μl NaPO_4_, pH 7.4; 100 μl 16% paraformaldehyde) [99]. The solution was incubated in the dark at room temperature for 30 minutes followed by 30 minutes at 4°C. After the fixative was removed, cells were washed three times with PBS before suspension in 500 μl PBS and incubation overnight in the dark at 4°C to allow for fluorophore maturation. Slides for microscopy were prepared by placing 10 μl of concentrated culture onto a thin layer of 1% agarose applied directly to the surface of the slide. Microscopy was performed using a 60x oil immersion Nikon Plan Apo objective on a Keyence BZ-X810 equipped with Chroma 49005-UF1 for RFP detection.

### Generation of strains

To generate *rho* expression plasmids, wild-type and mutant *rho* alleles were amplified from genomic DNA of *recV flg* OFF (RT1693) bacteria and 6 selected motile suppressors by PCR using primers R2308 and R2307 [96]. PCR products were cloned via the EcoRV and BamHI sites in pRT1611, a derivative of pRPF185 in which the *gusA* reporter gene was removed [33, 58]. After transformation into *E*. *coli* DH5α, Cm-resistant clones were recovered at 30°C to hinder additional mutations in *rho*. The presence of the *rho* insert and its sequence integrity were confirmed using primers R2308, R2307, R2366, and R2367. The expression plasmids and the pRT1611 control were introduced into *C*. *difficile* strains RT1693 (*recV flg* OFF), RT1702 (*recV flg* ON), RT1709 (MS5), and RT1941 (MS10) via conjugation with *E*. *coli* HB101(pRK24). The presence of the expected plasmid was confirmed by PCR with vector-specific primers R1832 and R1833.

Plasmids containing transcriptional fusions of the *phoZ* alkaline phosphatase gene to *flgB* and iterations of the upstream regulatory region were created previously [33]. These plasmids were introduced into heat-shocked RT1693, RT1702, MS5, and MS10 [101]. For fluorescence microscopy, pRT1676, a pDSW1728 derivative carrying P_*flgM*_::mCherryOpt [33], was introduced into RT1693, RT1694, MS5, and MS10 by conjugation with *E*. *coli* HB101(pRK24) and confirmed by PCR.

To introduce *C*. *difficile rho* (*Cd*-*rho*) into *B*. *subtilis* BS49, *Cd*-*rho* including its native ribosomal binding site (RBS) was amplified from R20291 genomic DNA by PCR using R2656 and R2657, digested with HindIII and SphI, and ligated into similarly digested pDR111, which allows for integration at the *amyE* site [102]. The resulting plasmid was transformed into *B*. *subtilis* BS49 strains bearing previously described transcriptional fusions of *phoZ* to *flgB* and its upstream regulatory region, and transformants were selected on LB-Spec agar.

### Alkaline phosphatase assays

Overnight (~16 h) cultures of *B*. *subtilis* BS49 and *C*. *difficile phoZ* reporter strains were diluted 1:50 (*C*. *difficile*) or 1:100 (*B*. *subtilis*) into BHIS medium. Thiamphenicol was added to *C*. *difficile* growth media for plasmid maintenance. To induce expression of *Cd-rho* in *B*. *subtilis*, 0.5 mM isopropyl β-D-1-thiogalactopyranoside (IPTG) was added to the growth medium when cultures reached OD_600_ ~0.3. Controls without induction were processed in parallel. Where indicated, bicyclomycin (Cayman Chemical) was used at 50 μg/mL. Mid-exponential phase cells (OD_600_ 0.8–1.3, 1 mL) were collected by centrifugation, the supernatant was discarded, and pellets were stored at -20°C overnight. Frozen pellets were thawed on ice, and the alkaline phosphatase (AP) assay was performed as previously described [64].

### Spore purification

Overnight cultures (100 μL) were plated on ten 70:30 agar plates [103]. After 72 hours of growth at 37°C, bacterial growth was scraped, suspended in 10 mL DPBS (Gibco) and kept at room temperature overnight. Spores were purified by collection of the growth in DPBS, then washing of the suspension four times with DPBS before purification using a sucrose gradient as described [104]. After discarding supernatant containing cell debris, the spore pellet was washed five more times with DPBS + 1% BSA. Spores were stored at room temperature until use.

### Germination assay

Spore germination was analyzed at room temperature (27°C) by measuring the change in OD_600_ [75]. Germination was carried out in clear 96-well flat bottom plates (Corning) in a final volume of 100 μl and final concentration of 30 mM glycine, 50 mM Tris, 100 mM NaCl, pH 7.5. Spores were heat-activated at 65°C for 30 minutes, cooled on ice and suspended to a final OD_600_ of 0.7. At the initiation of the experiment, 10 mM sodium taurocholate (Sigma Aldrich) (TA) was added to induce germination; no-taurocholate controls were done in parallel. Optical density at 600 nm was measured every 2 minutes for 1 hour using a BioTek Synergy plate reader.

### Sporulation assay

Sporulation assays were performed as described previously [74]. Briefly, *C*. *difficile* cultures were grown overnight in BHIS broth supplemented with 0.1% TA and 0.2% fructose to prevent spore accumulation. Cultures were diluted 1:30 in BHIS-0.1% TA-0.2% fructose, grown to an OD_600_ of 0.5 and 250 μl of culture applied to 70:30 agar as a lawn [103]. A control ethanol resistance sporulation assay was performed at this point to ensure no spores were present in exponential phase cultures. After 24 hours of incubation at 37°C, cells were suspended in BHIS to an OD_600_ of 1.0, and an ethanol resistance sporulation assay was performed. A 0.5 ml aliquot was mixed with 0.5 ml of 57% ethanol to achieve a final concentration of 28.5% ethanol, vortexed, and incubated for 15 minutes to eliminate all vegetative cells. Serial dilutions were made in PBS-0.1% TA and plated on BHIS-0.1% TA agar for spore enumeration. Vegetative cells were enumerated by plating serial dilutions of the BHIS cell suspension on BHIS agar. Sporulation efficiency was calculated as the total number of spores divided by the total number of viable cells (spores plus vegetative).

### Ethics statement

All animal studies were done in compliance with protocols approved by the UNC-CH Institutional Animal Care and Use Committee.

### Animal experiments

Groups of eight- to ten-week-old female and male C57BL/6 mice (Charles River) were given a cocktail of antibiotics in their drinking water provided *ab libitum* for 3 days [105]. The antibiotic cocktail consisted of kanamycin (400 mg/L), gentamicin (35 mg/L), colistin (850,000 units/L), vancomycin (45 mg/L), and metronidazole (215 mg/L) [105]. Four days prior to inoculation, the mice were switched to regular water for the remainder of the experiment. Clindamycin (10 μg/g body weight) was administered by intraperitoneal injection 48 hours prior to infection [14]. Mice were inoculated with 10^5^ spores by oral gavage; control mice received PBS only. Inoculums were quantified by plating serial dilution on BHIS-0.1% TA agar and enumerating CFU. The animals were subsequently monitored for weight loss and diarrheal disease. Fecal samples were collected in pre-weighed tubes every 24 hours for 9 days. Fecal pellets were suspended in 1 mL DPBS and heated at 55°C for 30 minutes. Serial dilutions were plated on TCCFA to enumerate CFU per gram feces [14]. Two independent experiments were done, each with 3 male and 3 female mice per *C*. *difficile* strain tested, for a total of 12 mice inoculated with each strain.

## Supporting information

S1 Data Single nucleotide polymorphisms identified in the MS1-7 and *recV flg* OFF strains compared to the R20291 reference genome. (XLS)

S1 Table Oligonucleotides used in this study. (DOCX)

S2 Table Complete analysis of single nucleotide polymorphisms (SNPs) in MS1-7 and *recV flg* OFF parent. (DOCX)

S3 Table Strains and plasmids used in this study. (DOCX)

S1 Fig *recV flg* OFF strains differ in status of *cwpV* switch. Orientation-specific PCR for the *cwpV* switch in *recV flg* OFF strains RT1693 and RT1694. Band sizes– 469bp (OFF) or 322bp (ON).
(TIF)

S2 Fig All 14 motile suppressors have restored motility. Quantification of swimming motility assays for the 14 MS, RT1693 (*recV flg* OFF), and RT1702 (*recV flg* ON). A non-motile *sigD* mutant was included as a control. The means and standard deviation of four biological replicates are shown.
(TIF)

S3 Fig Motile suppressors have altered growth. Growth curves of MS1-14, *recV flg* ON (RT1702), the *recV flg* OFF parents (values combined for RT1694 (parent of MS1-7) and RT1693 (parent of MS8-14)). Shown are the means and standard deviation for 3 biological replicates.
(TIF)

S4 Fig Expression of wild-type *rho* in MS5 and MS10 restores growth. Growth curves of MS5 and MS10 expressing wild-type *rho* (pRho) or bearing vector. The *recV flg* OFF and *recV flg* ON strains carrying vector were included. Expression of *rho* was induced with 10 ng/mL anhydrotetracycline (ATc). The means and standard deviation of three biological replicates are shown.
(TIF)

S5 Fig Rho does not affect flagellar gene expression at the level of promoter. An alkaline phosphatase (*phoZ*) reporter fusion to the *flgB* promoter (P_*flgB*_) and a promoterless:: *phoZ* construct were introduced into *recV flg* ON (RT1702), *recV flg* OFF (RT1693), MS5, and MS10. The means and standard deviation of 5 biological replicates are shown.
(TIF)

S6 Fig Supporting data for animal studies. (A) Orientation-specific PCR for the 6 additional invertible sequences found in R20291, in *recV flg* OFF (RT1693), MS5, and MS10. WT R20291 was included as a control. Orientation is labelled as ON/OFF for the three invertible sequences whose regulation has been studied (*cwpV*, *flg*, *cmrRST*) or as published (pub) or inverse (inv) based on the R20291 reference genome for the Cdi2, Cdi3, and Cdi5 sequences whose effects on gene expression are not known. (B) Antibiotic-treated male and female C57BL6 mice were inoculated with 10^5^ spores of the indicated *C*. *difficile* strain. CFU in feces collected every 24 hours post inoculation were enumerated as an indication of intestinal burden of *C*. *difficile*. Shown are the full courses of infection for two independent experiments that each included 3 male and 3 female mice. The data are separated by motile suppressor and its respective parent strain for clarity with the same data for wildtype R20291 in both upper and lower panels, with means and standard deviation shown. Dotted line represents a limit of detection.
(TIF)

S7 Fig Rho is dispensable for *C*. *difficile* spore germination. Purified spores of indicated strains were germinated in the presence of taurocholate (+) or in buffer without germinant as a control (-), and optical density (OD600) was measured. Germination was plotted as the ratio of optical density (OD600) at a given time point (tx) versus initial OD600 (t0). A representative germination plot of four independent experiments is shown.
(TIF)
