# Supplementary material for: Rho factor mediates flagellum and toxin phase variation and impacts virulence in Clostridioides difficile
Source: PLoS Pathog. 2020 Aug 12;16(8):e1008708. doi: 10.1371/journal.ppat.1008708 (PMC7446863; doi:10.1371/journal.ppat.1008708)
Supplement: S1 Table — (DOCX) [file ppat.1008708.s002.docx]

S1 Table. Oligonucleotides used in this study

| **Lab**  **Notation** | **Primer name** | **Sequence (5’ to 3’)**a | **Reference** |
| --- | --- | --- | --- |
| R850 | rpoCqF | CTAGCTGCTCCTATGTCTCACATC | [1] |
| R851 | rpoCqR | CCAGTCTCTCCTGGATCAACTA | [1] |
| R856 | flgBqF | GCAACTAATCTAAGAAGTCAGACAATAGC | [1] |
| R857 | flgBqR | AGGCATAGCATCATTTAGTGTTTCTTC | [1] |
| R1669 | flgMqF | AAGGAAATGGCAAGTGTG | [2] |
| R1670 | flgMqR | TTATCCTCGCATATCCTCT | [2] |
| R1854 | CDR20291_0240qF  (*fliC*) | CAAAGTAAGTCTATGGAGAA | [2] |
| R1855 | CDR20291_0240qR  (*fliC*) | ACAGATATACCATCTTGAAC | [2] |
| R908 | tcdRqF | AGCAAGAAATAACTCAGTAGATGATT | [1] |
| R909 | tcdRqR | TTATTAAATCTGTTTCTCCCTCTTCA | [1] |
| R852 | tcdAqF | GGAGAAGTCAGTGATATTGCTCTTG | [1] |
| R853 | tcdAqR | CAGTGGTAGAAGATTCAACTATAGCC | [1] |
| R854 | tcdBqF | AAGGAATATCTAGTTACAGAAGTATTAGAGC | [1] |
| R855 | tcdBqR | GCAGTGTCATTTATTTGACCTCCA | [1] |
| R1614 | CDR20291_0248InvF | AGGCAACTTTATAAAGAAATATTTAAATTTATATTAAAATATT TTTATATTTTTATTAGG | [2] |
| R1615 | CDR20291_0248InvR | CCTAATAAAAATATAAAAATATTTTAATATAAATTTAAATATTT CTTTATAAAGTTGCCT | [2] |
| R2307 | R202_rho_R | CAAGGATCCTCCATATTTGCATTTGTATTATTGATT | This work |
| R2308 | R202_rho_F2 | CAAGATATCCATGCCTTCAATTTCCAAAATAAG | This work |
| R2366 | CDR20291_3324intF | GGAAACTAATAGAAATGAAATAGCT | This work |
| R2367 | CDR20291_3324intR | AGCTATTTCATTTCTATTAGTTTCC | This work |
| R2656 | Rho_Gibson_F | GTGAGCGGATAACAATTAAGCTTCATGCCTTCAATTTCCAA AATAAG | This work |
| R2657 | Rho_Gibson_R | TCCACCGAATTAGCTTGCATGCATATAATCCATATTTGCATT TGTATTATTGATTTTAC | This work |
| R1832 | pRPF185MCS_F | TATTTCGATGCCCTGGACT | [3] |
| R1833 | pRPF185MCS_R | ATCCCCTACTACTGACAGCTTC | [3] |
| R1050 | cwpV_R | AGCATCTGCTATAGATGAGTCGTTT | [2] |
| R1920 | cwpV_pubF | CAAAACCATGTTTTTTATAACAATTCATTAAC | [2] |
| R1921 | cwpV_invF | GTTAATGAATTGTTATAAAAAACATGGTTTTG | [2] |
| R2064 | CDR20291_0685_R | GTTAAAAATTTAAGATATCTTTTCAGTATAATGGA | [4] |
| R2065 | CDR20291_0685_invF | CATTTCTAAGAAATATCCTAACATAAAAACAAAA | [4] |
| R2195 | CDR20291_0685_pubF | TTTTGTTTTTATGTTAGGATATTTCTTAGAAATG | [4] |
| R2067 | CDR20291_0963_R | CTACTGAATATCATATTAGACTCCTTTCTTAACCA | [4] |
| RT2068 | CDR20291_0963_pubF | GTAAATTAAGATGTATTTCATTTCTCAAAAATATCCT | [4] |
| R2196 | CDR20291_0963_invF | AGGATATTTTTGAGAAATGAAATACATCTTAATTTAC | [4] |
| R2070 | cmrRST_R | GGAGATATATGGAGTTAGTGGTGCAA | [4] |
| R2071 | cmrRST_invF | CTAGCCAATAGACAAGTTTCTAGAAAAATA | [4] |
| R2197 | cmrRST_pubF | TATTTTTCTAGAAACTTGTCTATTGGCTAG | [4] |
| R1455 | CDR20291_1514R | CAAGAATTCCAAGTTGAAAGTGAGATAC | [4] |
| R1737 | CDR20291_1514invF | CAGACAATTCAAACAAAAATAATC | [4] |
| R1738 | CDR20291_1514pubF | GATTATTTTTGTTTGAATTGTCTG | [4] |

a Restriction sites used for cloning are underlined

**References**

1. McKee, R.W., et al., *The second messenger cyclic di-GMP regulates Clostridium difficile toxin production by controlling expression of sigD.* Journal of Bacteriology, 2013. **195**(22): p. 5174-5185.
2. Anjuwon-Foster, B.R. and R. Tamayo, *A genetic switch controls the production of flagella and toxins in Clostridium difficile.* PLoS Genetics, 2017. **13**(3): p. e1006701.
3. Anjuwon-Foster, B.R., N. Maldonado-Vazquez, and R. Tamayo, *Characterization of flagellar and toxin phase variation in Clostridiodes difficile ribotype 012 isolates.* Journal of Bacteriology, 2018.
4. Sekulovic, O., et al., *Genome-wide detection of conservative site-specific recombination in bacteria.* PLoS Genetics, 2018. **14**(4): p. e1007332.
