# Supplementary figures and images for: Rho factor mediates flagellum and toxin phase variation and impacts virulence in Clostridioides difficile
Source: PLoS Pathog. 2020 Aug 12;16(8):e1008708. doi: 10.1371/journal.ppat.1008708 (PMC7446863; doi:10.1371/journal.ppat.1008708)

**S2 Table. Motile suppressor sequence analysis**
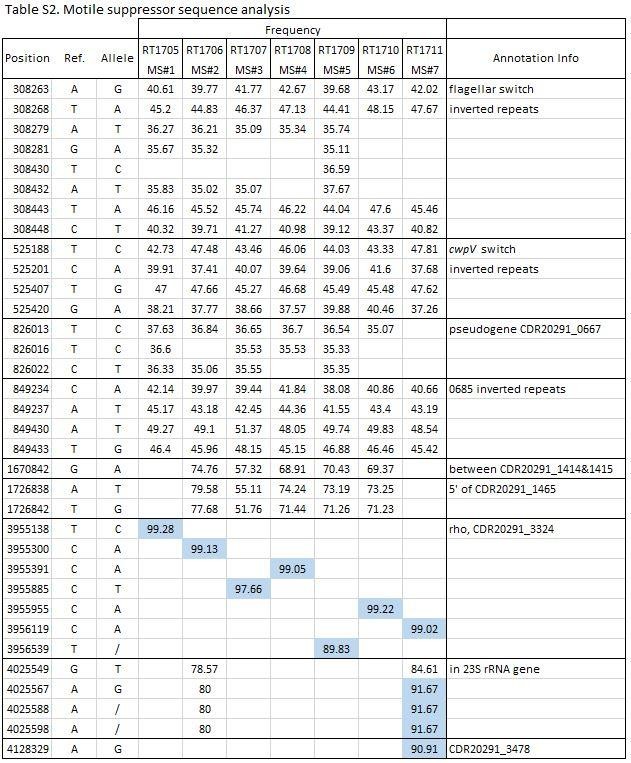

Supplement: S2 Table — (DOCX) [file ppat.1008708.s003.docx]

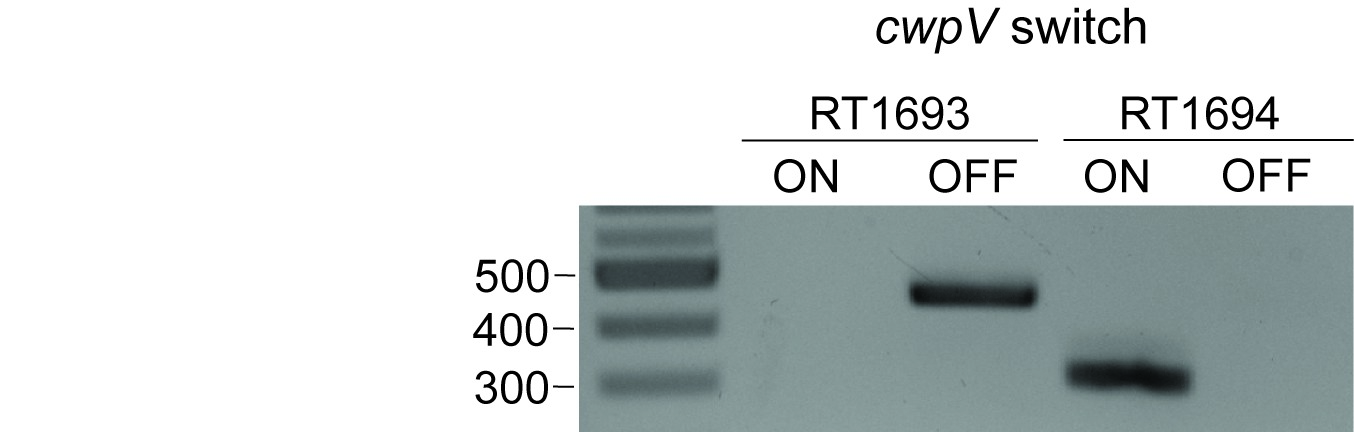

Supplement: S1 Fig — Orientation-specific PCR for the cwpV switch in recV flg OFF strains RT1693 and RT1694. Band sizes– 469bp (OFF) or 322bp (ON). (TIF) [file ppat.1008708.s005.tif]

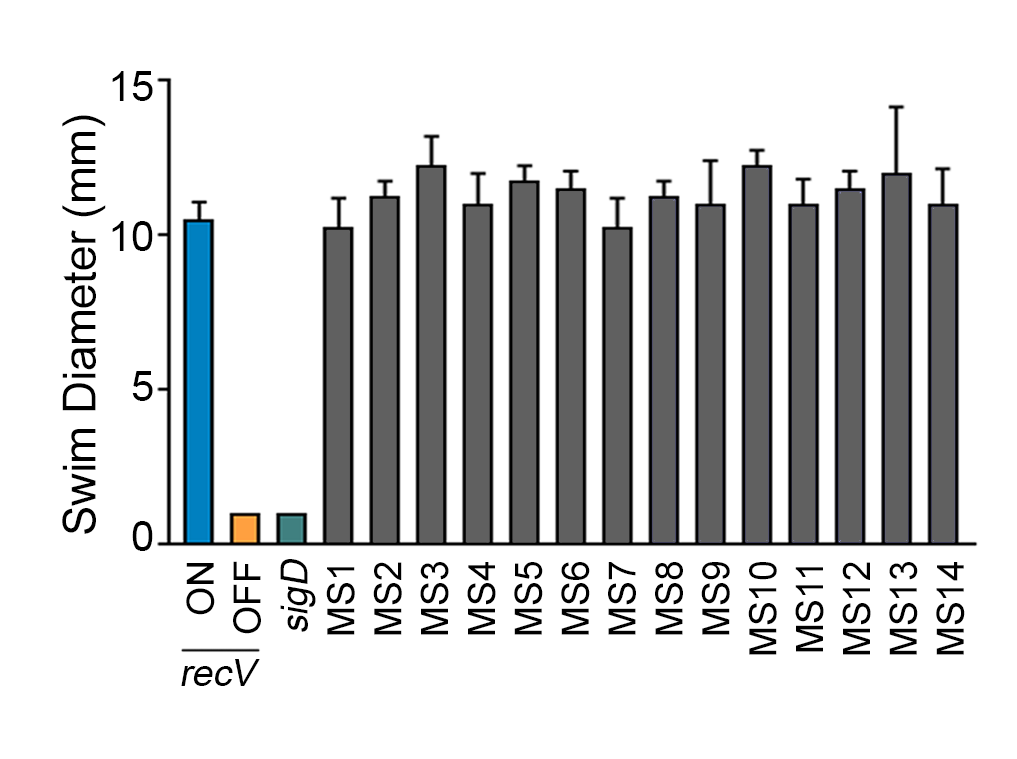

Supplement: S2 Fig — Quantification of swimming motility assays for the 14 MS, RT1693 (recV flg OFF), and RT1702 (recV flg ON). A non-motile sigD mutant was included as a control. The means and standard deviation of four biological replicates are shown. (TIF) [file ppat.1008708.s006.tif]

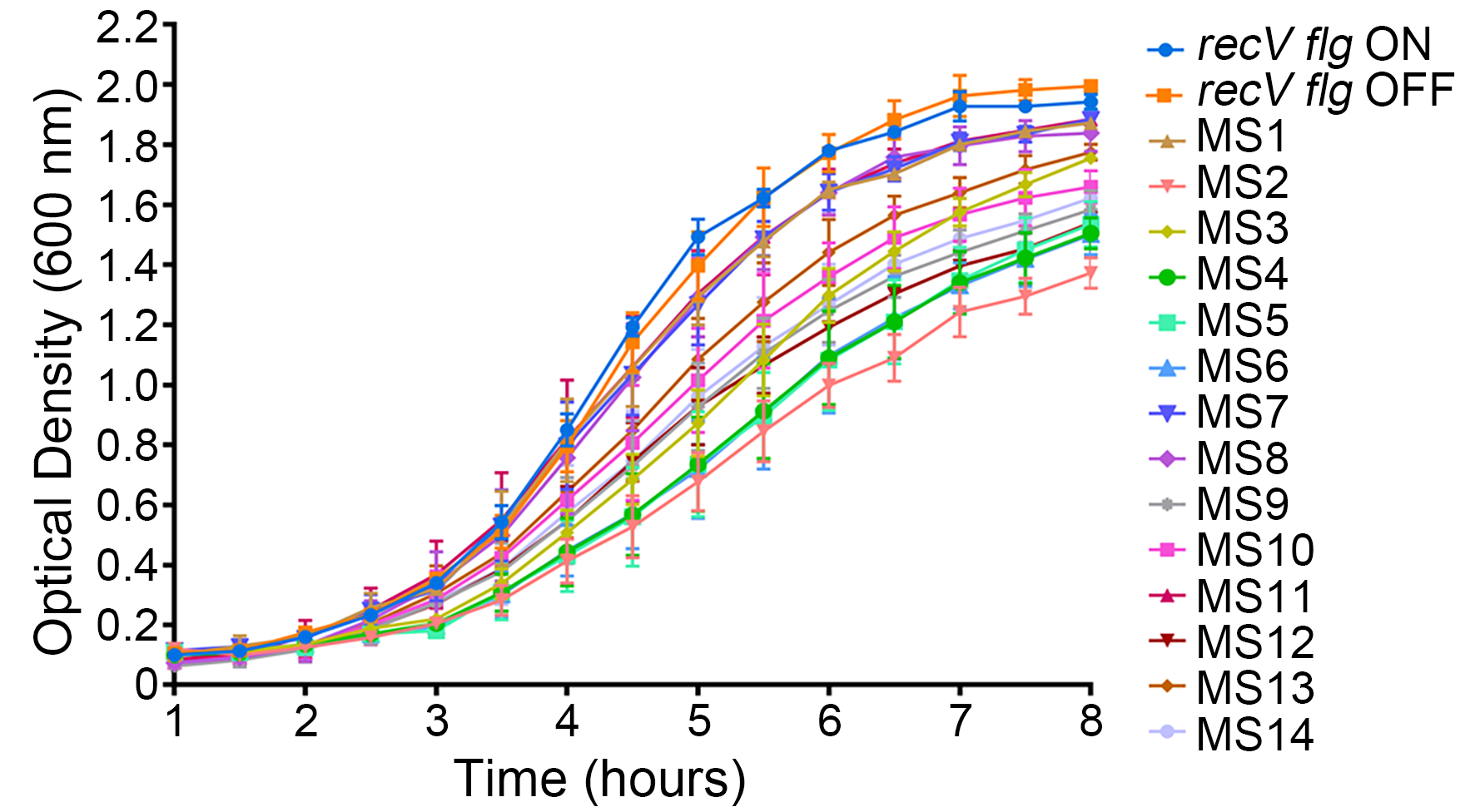

Supplement: S3 Fig — Growth curves of MS1-14, recV flg ON (RT1702), the recV flg OFF parents (values combined for RT1694 (parent of MS1-7) and RT1693 (parent of MS8-14)). Shown are the means and standard deviation for 3 biological replicates. (TIF) [file ppat.1008708.s007.tif]

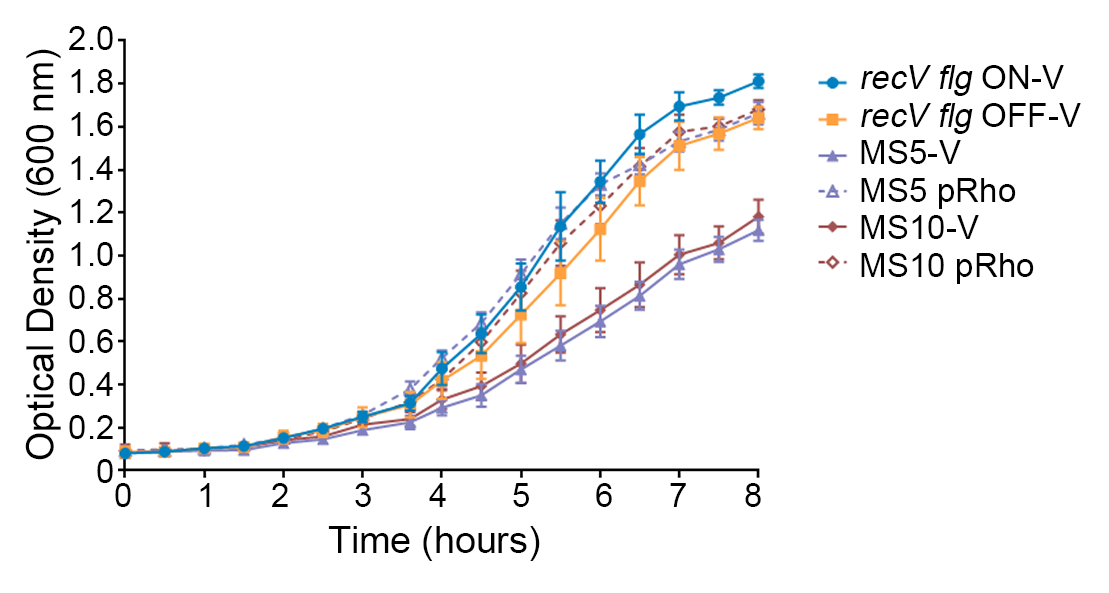

Supplement: S4 Fig — Growth curves of MS5 and MS10 expressing wild-type rho (pRho) or bearing vector. The recV flg OFF and recV flg ON strains carrying vector were included. Expression of rho was induced with 10 ng/mL anhydrotetracycline (ATc). The means and standard deviation of three biological replicates are shown. (TIF) [file ppat.1008708.s008.tif]

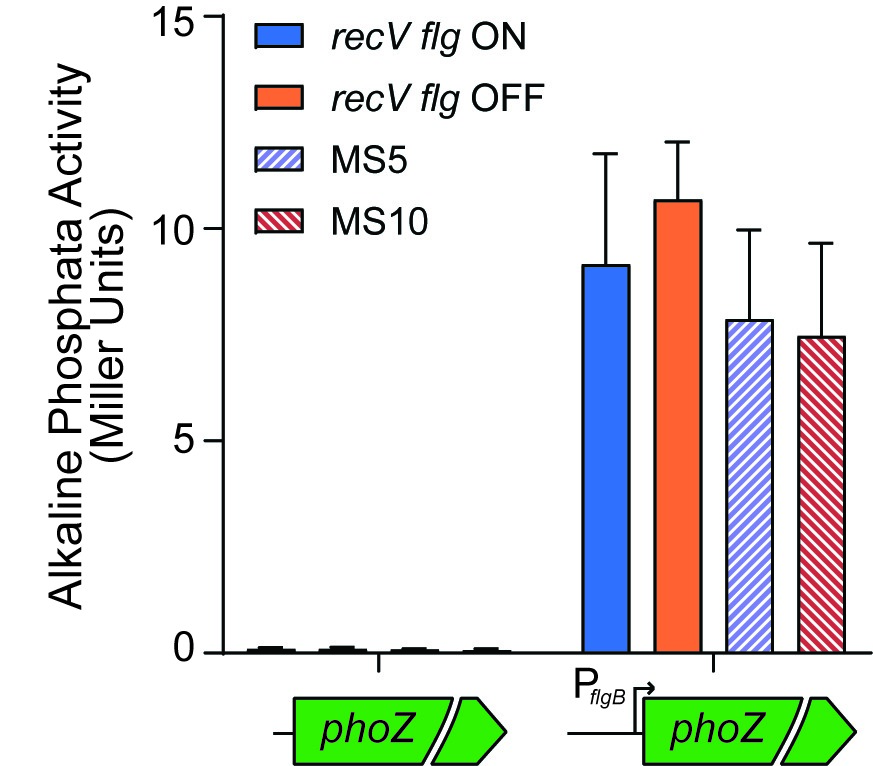

Supplement: S5 Fig — An alkaline phosphatase (phoZ) reporter fusion to the flgB promoter (PflgB) and a promoterless:: phoZ construct were introduced into recV flg ON (RT1702), recV flg OFF (RT1693), MS5, and MS10. The means and standard deviation of 5 biological replicates are shown. (TIF) [file ppat.1008708.s009.tif]

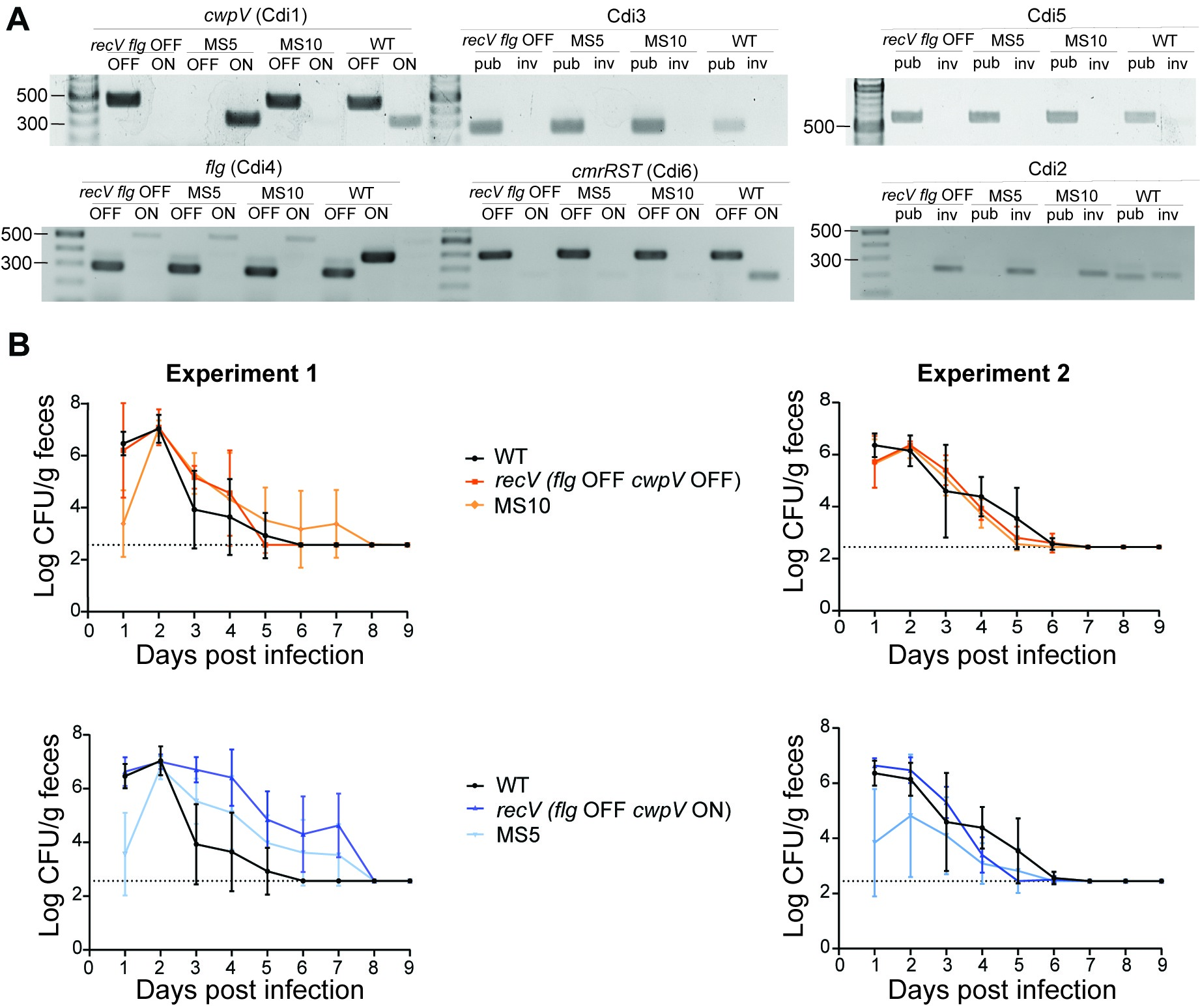

Supplement: S6 Fig — (A) Orientation-specific PCR for the 6 additional invertible sequences found in R20291, in recV flg OFF (RT1693), MS5, and MS10. WT R20291 was included as a control. Orientation is labelled as ON/OFF for the three invertible sequences whose regulation has been studied (cwpV, flg, cmrRST) or as published (pub) or inverse (inv) based on the R20291 reference genome for the Cdi2, Cdi3, and Cdi5 sequences whose effects on gene expression are not known. (B) Antibiotic-treated male and female C57BL6 mice were inoculated with 105 spores of the indicated C. difficile strain. CFU in feces collected every 24 hours post inoculation were enumerated as an indication of intestinal burden of C. difficile. Shown are the full courses of infection for two independent experiments that each included 3 male and 3 female mice. The data are separated by motile suppressor and its respective parent strain for clarity with the same data for wildtype R20291 in both upper and lower panels, with means and standard deviation shown. Dotted line represents a limit of detection. (TIF) [file ppat.1008708.s010.tif]

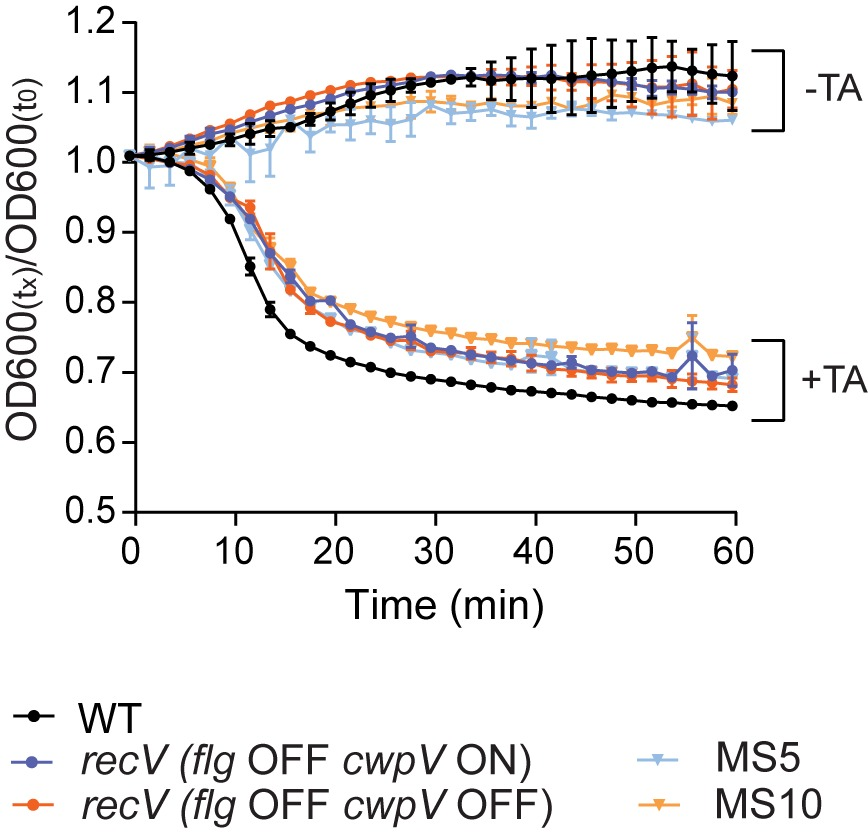

Supplement: S7 Fig — Purified spores of indicated strains were germinated in the presence of taurocholate (+) or in buffer without germinant as a control (-), and optical density (OD600) was measured. Germination was plotted as the ratio of optical density (OD600) at a given time point (tx) versus initial OD600 (t0). A representative germination plot of four independent experiments is shown. (TIF) [file ppat.1008708.s011.tif]
