# Supplementary material for: Rho factor mediates flagellum and toxin phase variation and impacts virulence in Clostridioides difficile
Source: PLoS Pathog. 2020 Aug 12;16(8):e1008708. doi: 10.1371/journal.ppat.1008708 (PMC7446863; doi:10.1371/journal.ppat.1008708)
Supplement: S3 Table — (DOCX) [file ppat.1008708.s004.docx]

S3 Table. Strains and plasmids used in this study

| **Lab Notation** | **Strain/Plasmid Name** | **Description** | **Reference** |
| --- | --- | --- | --- |
|  | *Escherichia coli* DH5α | F- φ80*lacZ*ΔM15 Δ(*lacZY*A-*argF*)U169 *recA1 endA1*  *hsdR*17(rκ -, mκ+) *phoA supE44 thi*-1 *gyrA96 relA1* λ- *tonA* | Invitrogen [1] |
| RT270 | *Escherichia coli*  HB101(pRK24) | *E. coli* used in conjugations with *C. difficile*, ApR, CmR | [2] |
| RT275 | *B. subtilis* BS49 | Derived from *B. subtilis* 168, CU2189::Tn916 | [3] |
| RT273 | *C. difficile* R20291 | Ribotype 027 strain | [4] |
| RT1566 | *sigD* | R20291 *sigD*::*ermB* | [5] |
| RT1702 | *recV flg* ON | R20291 *recV::ermB* (*flg* ON; *cwpV* OFF) | [5] |
| RT1693 | *recV flg* OFF *cwpV* ON | R20291 *recV::ermB* (*flg* OFF; *cwpV* ON) | [5, 6] |
| RT1694 | *recV flg* OFF *cwpV* OFF | R20291 *recV::ermB* (*flg* OFF; *cwpV* OFF) | [5, 6] |
| RT1705 | MS1 | R20291 *recV flg* OFF motile suppressor, *rho*-N533S | This work |
| RT1706 | MS2 | R20291 *recV flg* OFF motile suppressor, *rho*-G479V | This work |
| RT1707 | MS3 | R20291 *recV flg* OFF motile suppressor, *rho*-G284E | This work |
| RT1708 | MS4 | R20291 *recV flg* OFF motile suppressor, *rho*-E449-Stop | This work |
| RT1709 | MS5 | R20291 *recV flg* OFF motile suppressor, *rho*-N66-FS | This work |
| RT1710 | MS6 | R20291 *recV flg* OFF motile suppressor, *rho*-E261-Stop | This work |
| RT1711 | MS7 | R20291 *recV flg* OFF motile suppressor, *rho*-R206I | This work |
| RT1939 | MS8 | R20291 *recV flg* OFF motile suppressor, *rho*-S478F | This work |
| RT1940 | MS9 | R20291 *recV flg* OFF motile suppressor, *rho*-L409P | This work |
| RT1941 | MS10 | R20291 *recV flg* OFF motile suppressor, *rho*-E113-Stop | This work |
| RT1942 | MS11 | R20291 *recV flg* OFF motile suppressor, WT rho allele | This work |
| RT1943 | MS12 | R20291 *recV flg* OFF motile suppressor, *rho*-R322I | This work |
| RT1944 | MS13 | R20291 *recV flg* OFF motile suppressor, *rho*-D320A | This work |
| RT1945 | MS14 | R20291 *recV flg* OFF motile suppressor, *rho*-E247-Stop | This work |
| RT1615 | *recV flg* ON-V | RT1702 with pRT1611 | This work |
| RT2198 | *recV flg* OFF-V | RT1693 with pRT1611 | This work |
| RT2262 | *sigD*-V | RT1566 with pRT1611 | This work |
| RT2260 | MS5-V | RT1709 with pRT1611 | This work |
| RT2258 | MS5 pRho | RT1709 with pRT1611::*rho* (CDR20291_3324) | This work |
| RT2261 | MS10-V | RT1941 with pRT1611 | This work |
| RT2259 | MS10 pRho | RT1941 with pRT1611::*rho* (CDR20291_3324) | This work |
| RT1904 | *recV flg* OFF *rho*WT | RT1693 with pRT1611::*rho* | This work |
| RT1905 | *recV flg* OFF N533S | RT1693 with pRT1611::*rho*-N533S | This work |
| RT1907 | *recV flg* OFF G284E | RT1693 with pRT1611::*rho*-G284E | This work |
| RT1909 | *recV flg* OFF N66-FS | RT1693 with pRT1611::*rho*-N66-FS | This work |
| RT1910 | *recV flg* OFF E261-Stop | RT1693 with pRT1611::*rho-*E261-Stop | This work |
| RT1911 | *recV flg* OFF R206I | RT1693 with pRT1611::*rho*-R206I | This work |
| RT2207 | *recV flg* OFF E113-Stop | RT1693 with pRT1611::*rho*-E113-Stop | This work |
| RT1838 | *recV flg* OFF::*phoZ* | RT1693 with pRT1824 ::*phoZ* | This work |
| RT1839 | *recV flg* OFF P*flgB*::*phoZ* | RT1693 with pRT1824: P*flgB*::*phoZ* | This work |
| RT1840 | *recV flg* OFF P*flgB*-  5'UTRON*::phoZ* | RT1693 with pRT1824: P*flgB*-5'UTRON-*flgB::phoZ* | This work |
| RT1841 | *recV flg* OFF P*flgB*- 5'UTROFF*::phoZ* | RT1693 with pRT1824: P*flgB*-5'UTROFF-*flgB::phoZ* | This work |
| RT1842 | *recV flg* ON::*phoZ* | RT1702 with pRT1824: ::*phoZ* | This work |
| RT1843 | *recV flg* ON P*flgB*::*phoZ* | RT1702 with pRT1824: P*flgB*::*phoZ* | This work |
| RT1844 | *recV flg* ON P*flgB*-  5'UTRON*::phoZ* | RT1702 with pRT1824: P*flgB*-5'UTRON-*flgB::phoZ* | This work |
| RT1845 | *recV flg* ON P*flgB*-  5'UTROFF*::phoZ* | RT1702 with pRT1824: P*flgB*-5'UTROFF-*flgB::phoZ* | This work |
| RT2299 | MS5 ::*phoZ* | RT1709 with pRT1824: ::*phoZ* | This work |
| RT2300 | MS5 P*flgB*::*phoZ* | RT1709 with pRT1824: P*flgB*::*phoZ* | This work |
| RT2323 | MS5 P*flgB*-5'UTRON*::phoZ* | RT1709 with pRT1824: P*flgB*-5'UTRON-*flgB::phoZ* | This work |
| RT2324 | MS5 P*flgB*-5'UTROFF*::phoZ* | RT1709 with pRT1824: P*flgB*-5'UTROFF-*flgB::phoZ* | This work |
| RT2314 | MS10 ::*phoZ* | RT1941 with pRT1824::*phoZ* | This work |
| RT2315 | MS10 P*flgB*::*phoZ* | RT1941 with pRT1824::P*flgB*::*phoZ* | This work |

| RT2316 | MS10 P*flgB*-5'UTROFF*::phoZ* | RT1941 with pRT1824::P*flgB*-5'UTROFF-*flgB::phoZ* | This work |
| --- | --- | --- | --- |
| RT2317 | MS10 P*flgB*-5'UTRON*::phoZ* | RT1941 with pRT1824::P*flgB*-5'UTRON-*flgB::phoZ* | This work |
| RT1695 | R20291 P*flgM*::*mCherryOpt* | RT273 with pDSW1728: P*flgM::mCherryOpt* | [5] |
| RT2485 | *recV flg* OFF  P*flgM*::*mCherryOpt* | RT1693 with pDSW1728: P*flgM*::*mCherryOpt* | This work |
| RT2486 | *recV flg* OFF *cwpV* OFF  P*flgM*::*mCherryOpt* | RT1694 with pDSW1728: P*flgM*::*mCherryOpt* | This work |
| RT2487 | MS5 P*flgM*::*mCherryOpt* | RT1709 with pDSW1728: P*flgM*::*mCherryOpt* | This work |
| RT2488 | MS10 P*flgM*::*mCherryOpt* | RT1941 with pDSW1728: P*flgM*::*mCherryOpt* | This work |
| RT1393 | BS49 P*flgB::phoZ* | BS49 with R20291 P*flgB-*UTRON*-flgB::phoZ* reporter in  Tn916 | [5] |
| RT1394 | BS49 P*flgB-*UTRON*::phoZ* | BS49 with R20291 P*flgB-*UTRON*-flgB::phoZ* reporter in  Tn916 | [5] |
| RT1395 | BS49 P*flgB-*UTROFF*::phoZ* | BS49 with R20291 P*flgB-*UTROFF*-flgB::phoZ* reporter in  Tn916 | [5] |
| RT2393 | BS49 P*flgB::phoZ + Cd-rho* | RT1393 with R20291 *rho* in *amyE* site | This work |
| RT2394 | BS49 P*flgB-*UTROFF*::phoZ +*  *Cd-rho* | RT1395 with R20291 *rho* in *amyE* site | This work |
| RT2407 | BS49 P*flgB-*UTRON*::phoZ +*  *Cd-rho* | RT1394 with R20291 *rho* in *amyE* site | This work |
|  |  |  |  |
| **Plasmids** |  |  |  |
| **Lab**  **notation** | **Plasmid Name** | **Description** | **Citation** |
|  | pRPF185 | pMTL960-derivative, contains ATc-inducible P*tet* promoter | [7] |
|  | pRT1611 | *gusA* removed from pRPF185 | [5] |
|  | pRPF144 | pMTL960-derivative | [7] |
| pRT1824 | ::*phoZ* promoterless control | P*cwpV2*-*gusA* removed from pRPF144 and replaced with  ::*phoZ* | This work |
| pRT1825 | P*flgB*::*phoZ* | pRT1824::P*flgB*::*phoZ* | This work |
| pRT1826 | P*flgB*-5'UTRON-*flgB::phoZ* | pRT1824::P*flgB*-5'UTRON-*flgB::phoZ* | This work |
| pRT1827 | P*flgB*-5'UTROFF-*flgB::phoZ* | pRT1824::P*flgB*-5'UTROFF-*flgB::phoZ* | This work |
| pRT1676 | P*flgM*::*mCherryOpt* | pDSW1728: P*flgM*::*mCherryOpt* | [5] |
| pRT1882 | pRho | pRPF185::*rho* | This work |
| pRT1883 | pRho-N533S | pRPF185::*rho*-N533S | This work |
| pRT1885 | pRho-G284E | pRPF185::*rho*-G284E | This work |
| pRT1887 | pRho-N66-FS | pRPF185::*rho*-N66-FS | This work |
| pRT1888 | pRho-E261-Stop | pRPF185::*rho-*E261-Stop | This work |
| pRT1889 | pRho-R206I | pRPF185::*rho*-R206I | This work |
| pRT2101 | pRho-E113-Stop | pRPF185::*rho*-E113-Stop | This work |
| pRT2377 | pDR111 | Contains IPTG-inducible P*hyper-spank* promoter | E. Shank,[8] |
| pRT2391 | *Cd-rho* | pDR111::*rho* | This work |

**References**

1. Hanahan, D., *Studies on transformation of Escherichia coli with plasmids.* J Mol Biol, 1983. **166**(4): p. 557-80.
2. McBride, S.M. and A.L. Sonenshein, *Identification of a genetic locus responsible for antimicrobial peptide resistance in Clostridium difficile.* Infection and immunity, 2011. **79**(1): p. 167-176.
3. Browne, H.P., et al., *Complete genome sequence of BS49 and draft genome sequence of BS34A, Bacillus subtilis strains carrying Tn916.* FEMS Microbiol Lett, 2015. **362**(3): p. 1-4.
4. Stabler, R.A., et al., *Comparative genome and phenotypic analysis of Clostridium difficile 027 strains provides insight into the evolution of a hypervirulent bacterium.* Genome biology, 2009. **10**(9): p. R102.
5. Anjuwon-Foster, B.R. and R. Tamayo, *A genetic switch controls the production of flagella and toxins in Clostridium difficile.* PLoS Genetics, 2017. **13**(3): p. e1006701.
6. Sekulovic, O., et al., *The Clostridium difficile cell wall protein CwpV confers phase-variable phage resistance.*

Molecular Microbiology, 2015. **98**(2): p. 329-342.

1. Fagan, R.P. and N.F. Fairweather, *Clostridium difficile has two parallel and essential Sec secretion systems.* The Journal of biological chemistry, 2011. **286**(31): p. 27483-27493.
2. Ben-Yehuda, S., D.Z. Rudner, and R. Losick, *RacA, a bacterial protein that anchors chromosomes to the cell poles.* Science, 2003. **299**(5606): p. 532-6.
